# Rapid dynamics of electrophysiological connectome states are heritable

**DOI:** 10.1162/netn_a_00391

**Published:** 2024-12-10

**Authors:** Suhnyoung Jun, Thomas H. Alderson, Stephen M. Malone, Jeremy Harper, Ruskin H. Hunt, Kathleen M. Thomas, William G. Iacono, Sylia Wilson, Sepideh Sadaghiani

**Affiliations:** Department of Psychology, University of Illinois Urbana-Champaign, Champaign, IL, USA; Beckman Institute for Advanced Science and Technology, University of Illinois Urbana-Champaign, Champaign, IL, USA; Department of Psychology, University of Minnesota Twin Cities, Minneapolis, MN, USA; Institute of Child Development, University of Minnesota Twin Cities, Minneapolis, MN, USA; Neuroscience Program, University of Illinois Urbana-Champaign, Champaign, IL, USA

**Keywords:** Dynamic functional connectivity, Electrophysiology, Heritability, Variance component modeling, Twin study, Hidden Markov modeling

## Abstract

Time-varying changes in whole-brain connectivity patterns, or connectome state dynamics, are a prominent feature of brain activity with broad functional implications. While infraslow (<0.1 Hz) connectome dynamics have been extensively studied with fMRI, rapid dynamics highly relevant for cognition are poorly understood. Here, we asked whether rapid electrophysiological connectome dynamics constitute subject-specific brain traits and to what extent they are under genetic influence. Using source-localized EEG connectomes during resting state (*N* = 928, 473 females), we quantified the heritability of multivariate (multistate) features describing temporal or spatial characteristics of connectome dynamics. States switched rapidly every ∼60–500 ms. Temporal features were heritable, particularly Fractional Occupancy (in theta, alpha, beta, and gamma bands) and Transition Probability (in theta, alpha, and gamma bands), representing the duration spent in each state and the frequency of state switches, respectively. Genetic effects explained a substantial proportion of the phenotypic variance of these features: Fractional Occupancy in beta (44.3%) and gamma (39.8%) bands and Transition Probability in theta (38.4%), alpha (63.3%), beta (22.6%), and gamma (40%) bands. However, we found no evidence for the heritability of dynamic spatial features, specifically states’ Modularity and connectivity pattern. We conclude that genetic effects shape individuals’ connectome dynamics at rapid timescales, specifically states’ overall occurrence and sequencing.

## Author Summary

In this study, we investigate the genetic influence on rapid electrophysiological connectome dynamics. Using hidden Markov model on source-localized EEG data at rest, we obtained measures describing temporal trajectories and time-varying spatial characteristics of connectome states. Applying two heritability assessment methods to these multivariate, time-varying connectome dynamics features, we discovered that the duration (Fractional Occupancy) and frequency of state switches (Transition Probability) were heritable, particularly in theta, alpha, beta, and gamma bands. However, no genetic influence was observed on spatial features.

## INTRODUCTION

Investigations of the functional connectome have been dominated by fMRI due to its superb spatial resolution, which, however, does not allow the study of rapid connectome dynamics at subsecond, cognitively highly relevant timescales. A growing body of work has established that noninvasive, real-time methods, that is, EEG and Magnetoencephalography (MEG), can provide a window into the connectome and, importantly, its rapid dynamics (static/time-averaged connectome: (Brookes et al., 2011; Deligianni, Centeno, Carmichael, & Clayden, 2014; de Pasquale et al., 2010; Hipp & Siegel, 2015; Wirsich et al., 2017); connectome dynamics: (Baker et al., 2014; Brookes et al., 2014; Coquelet et al., 2022; Sitnikova et al., 2020; Wirsich, Giraud, & Sadaghiani, 2020)). EEG/MEG connectome studies capitalize on methodological advances that combine source localization with correction of source-leakage, an artifact arising from the activity of the same electrical source in the brain being picked up by multiple sensors. As such, real-time methods can extend our understanding of connectome dynamics beyond the knowledge gained from fMRI (for review, see Sadaghiani & Wirsich, 2020).

What we know from fMRI connectome studies suggests that the time-varying dynamics in large-scale connectivity are of functional importance for cognition (Cohen, 2018; Preti, Bolton, & Van De Ville, 2017). These reconfigurations can be characterized as flexible changes in connectome states, representing varying strengths of connectivity between specific sets of brain regions within the whole-brain connectome, which occur repeatedly over time. Such time-varying features have been linked to a wide range of behaviors and cognitive processes, encompassing both the temporal organization of connectome state transitions (Eichenbaum, Pappas, Lurie, Cohen, & D’Esposito, 2021; Jun, Alderson, Altmann, & Sadaghiani, 2022; Vidaurre, Smith, & Woolrich, 2017) and changes in the spatial organization of connectome states (Douw, Wakeman, Tanaka, Liu, & Stufflebeam, 2016; Sadaghiani, Poline, Kleinschmidt, & D’Esposito, 2015; Thompson et al., 2013). Particular temporal features of fMRI-derived connectome dynamics, specifically the proportion of the total recording time spent in each connectome state (Fractional Occupancy) and the probability to transition between specific pairs of connectome states (Transition Probability), have been linked to behavioral performance (Eichenbaum et al., 2021; Jun et al., 2022; Vidaurre et al., 2017) and found to be heritable (Jun et al., 2022; Vidaurre et al., 2017). More specifically, our previous fMRI work has established substantial genetic effects (*h*^2^ ∼ 40%) and behavioral relevance of Fractional Occupancy and Transition Probability (Jun et al., 2022). We further identified specific genetic polymorphisms predictive of fMRI-derived Fractional Occupancy and Transition Probability via the regulatory impact of modulatory neurotransmitter systems (see our pre-registered work: https://doi.org/10.17605/OSF.IO/VF2ZW).

However, as mentioned above, the temporal dynamics captured by the slow fMRI-derived indirect measure of neural activity, or BOLD signal, limit the study of rich, subsecond temporal dynamics. While direct electrophysiological techniques, that is, MEG or EEG, can capture such rapid dynamics (Baker et al., 2014; Coquelet et al., 2022; Quinn et al., 2018), their use in the study of individual differences and heritability has primarily focused on the electrophysiological power spectrum (D. J. A. Smit, Posthuma, Boomsma, & Geus, 2005; van Beijsterveldt, Molenaar, de Geus, & Boomsma, 1996) or *static* (time-averaged) connectivity (synchrony across less than 20 EEG electrodes (Chorlian et al., 2007; Posthuma et al., 2005; van Beijsterveldt, Molenaar, de Geus, & Boomsma, 1998); MEG power-envelope functional connectivity (FC; Colclough et al., 2017)). When investigating large-scale brain *dynamics* with MEG/EEG, one area of focus has been microstates, denoting recurrent, spatially diffuse sensor-level topographies that transition rapidly (every ∼40–200 ms) (Coquelet et al., 2022; Lehmann, Pascual-Marqui, & Michel, 2009). The temporal sequencing of these EEG microstates was found to exhibit scale-free dynamics (Gschwind, Michel, & Van De Ville, 2015; Van De Ville, Britz, & Michel, 2010), interpreted to reflect high flexibility in information processing critical for cognition. Further, the temporal characteristics of microstates have been associated with fluid intelligence (duration of microstates; Santarnecchi et al., 2017) and psychiatric disorders (temporal sequence and duration of microstates; see the review of Rieger, Hernandez, Baenninger, & Koenig, 2016). However, the topographies reflected in microstates are typically spatially diffused, because they are estimated in “sensor space” rather than on the cortical surface and, thus, fall short of informing about connectome states that encompass spatially localized coactivations across networks of brain regions. Moving to dynamics in *source-localized* EEG, we have recently shown that rapid temporal dynamics of recurrent connectome states are of functional significance, explaining individuals’ cognitive abilities (Jun et al., 2024). This individual specificity invites the question to what degree such rapid connectome state dynamics are heritable.

In the current study, we investigated whether temporal and spatial features of fast connectome dynamics in canonical electrophysiological frequency bands are attributable to genetic effects. To address this question, we applied the hidden Markov model (HMM) to extract discrete brain states using source-reconstructed resting-state EEG data from the Minnesota Twin Family Study (Keyes et al., 2009; Wilson et al., 2019). The dataset included monozygotic (MZ) and dizygotic (DZ) twin pairs and pairs of unrelated individuals. Consistent with our preceding heritability study in fMRI (Jun et al., 2022), we included two temporal features and two spatial features based on prior evidence for their behavioral relevance. Interestingly, only temporal features were found to be heritable in the fMRI study. Yet, we included the spatial features in the current study given that rapid EEG-derived dynamics might capture connectivity processes different from those observed in fMRI. The temporal features consisted of Fractional Occupancy and Transition Probability, and the spatial features included time-varying Modularity (Modularity_Time-Varying_) and the time-varying strength of FC (FC_Time-Varying_) of the set of connections (clusters) exhibiting the strongest cross-state change. Specifically, Modularity measures the balance between segregation (prioritizing processing within specialized networks) and integration (promoting interaction among different networks; Newman, 2006). We examined the effect of genetic relatedness on each multivariate connectome features and fitted quantitative genetic models to quantify the genetic effects. To the best of our knowledge, this is the first EEG study investigating the heritability of source-space connectome dynamics. This study sheds light on genetic contributions to individual differences in subsecond connectome dynamics.

## MATERIALS AND METHODS

**Figure 1.**
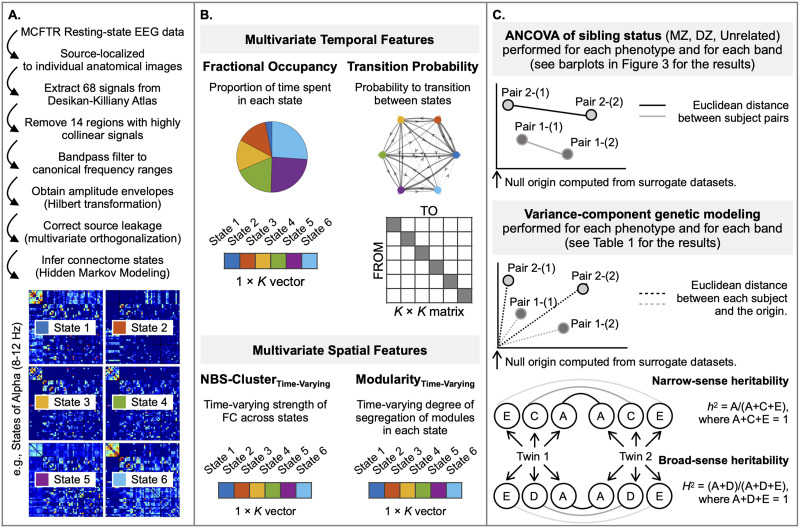
An overview of the analysis pipeline. (A) We used resting-state source-space EEG and structural images from the Minnesota Twin Family Study samples. We employed HMM to extract *K* = 6 discrete connectome states (or *K* = 4 states for replication) for each canonical EEG frequency band associated with a state time course for each subject indicating the probability of when each state is active. The amplitude coupling-based FC matrices of the states are shown at the bottom. The six states are color coded (blue, red, yellow, green, purple, and light blue) to illustrate their contribution to the connectome’s dynamic features of interest. (B) We constructed each feature in a multivariate manner to comprehensively represent all states. Specifically, multivariate temporal features were defined as the proportion of the recording time spent in each connectome state (Fractional Occupancy) and the probability matrix of transitioning between all possible pairs of discrete states (Transition Probability). Multivariate spatial features include Modularity_Time-Varying_ and FC_Time-Varying_ averaged across the set of connections (region-pairs) with the strongest dynamic changes across states (Zalesky, Fornito, & Bullmore, 2010). (C) We tested whether genetically more related subjects displayed greater similarity in their multivariate features than genetically less related subjects. First, for each feature of dimension *m*, we estimated a null model-derived origin point in the *m*-dimensional space. The position of each subject’s multidimensional feature value was estimated relative to this origin for genetic modeling. Further, the similarity of this position between a given pair of subjects was quantified as Euclidean distance for ANCOVA analyses; a one-way ANCOVA of the factor sibling status with three levels (MZ twins, sex-matched DZ twins, and sex-matched pairs of unrelated individuals) was performed on the distance value for each of the features. As a result, ANCOVA analysis was performed 20 times (i.e., four phenotypes and five frequency bands). Second, we employed structural equation modeling (i.e., genetic variance component model) to quantify the genetic effects. Phenotypic variance of a trait was partitioned into additive genetic (denoted A), common environmental (denoted C) or dominant genetic effect (denoted D), and unique environmental (denoted E) components. Narrow-sense heritability (*h*^2^) is quantified as the proportion of variance attributed to the genetic factor (A) and broad-sense heritability (*H*^2^) as the proportion of variance attributed to A and D factors. Therefore, the genetic variance component model was performed 20 times (i.e., four phenotypes and five frequency bands).

Figure 1 is a schematic representation of the overall approach and analysis subsections.

### Subjects

Participants for the present investigation are from the two independent cohorts of twins from the Minnesota Twin and Family Research (MCTFR; Iacono, Carlson, Taylor, Elkins, & McGue, 1999; Keyes et al., 2009; Wilson et al., 2019). Twins in both cohorts have been followed periodically since approximately the age of 11. As part of their most recent assessment, participants underwent structural MRI scans in addition to resting EEG recordings. At the time of initial recruitment and at each follow up, participants gave written informed consent or assent, if under the age of 18, for their participation.

From a total of 1,164 subjects in two independent cohorts of MCTFR twins completing virtually identical assessments, 928 had usable and complete EEG data that were source-localized successfully (see the EEG Signal Preprocessing and Source Localization section), thus permitting HMM-based estimation of discrete connectome states. The included subjects (473 females) were 23–40 years of age at time of data acquisition. Subsequently, 463 sex-matched pairs (926 subjects) were formed for heritability analysis as follows: 206 MZ twin pairs, 112 sex-matched DZ twin pairs, and 145 pairs of sex-matched unrelated individuals. Each subject entered only one pair. Note that the twins grew up together, and the unrelated individuals are twins whose co-twin lacked complete data and were, therefore, not part of the analytic sample. Further, all pairs of the unrelated individuals, except for two pairs (age difference: 9 and 17 years, respectively), were age-matched. In the following statistical analyses, age and sex were adjusted at all times.

### MRI and EEG Acquisition

Structural MRI data were collected on either 3 T Siemens Trio or Prisma MRI scanner (32-channel array head coil) at the Center for Magnetic Resonance Research, University of Minnesota. Three-dimensional T1-weighted sagittal plane anatomical images were acquired using the following magnetization-prepared rapid gradient echo sequence: TR = 2,530 ms; TE = 3.65 ms; flip angle = 7°; matrix size = 256 × 256; FOV = 256 mm; GRAPPA = 2; 240 coronal slices with 1-mm isotropic voxels; single shot; interleaved acquisition.

While recording EEG, participants rested comfortably in a darkened room, with their head and neck supported while hearing a 55-dB white noise played through headphones. They were instructed to keep their eyes closed and relax. A recorded voice subsequently instructed them to open the eyes or close them at 1-min intervals. A total of 6 min of EEG was collected, 3 min with eyes open and 3 min with eyes closed. EEG data were acquired from 61 scalp electrodes arranged according to the International 10/10 system using a BioSemi ActiveTwo system (BioSemi, Amsterdam, The Netherlands) at 1,024 Hz. ActiveTwo amplifiers are Direct-Coupled (DC) coupled. ActiveTwo signals are monopolar. They were low-pass filtered using a digital fifth-order Bessel antialiasing sinc filter with a cutoff frequency (3-dB attenuation) of 205 Hz. Pairs of electrodes placed above and below the right eye or on the outer canthus of each eye allowed for detecting blinks and other eye movements. Additional electrodes were placed on the left and right earlobes, and the average of these signals was derived offline to serve as a reference.

### EEG Signal Preprocessing and Source Localization

Raw resting-state EEG signals were preprocessed using a monitored automated pipeline (https://www.github.com/sjburwell/eeg_commander) of the Minnesota Twin Family Study group and EEGLAB (Delorme & Makeig, 2004) in MATLAB (version R2021b, MathWorks, Inc.). Signals were down-sampled to 256 Hz, filtered with a 0.1-Hz high-pass filter (*firfilt* EEGLAB plugin; 1,286 Kaiser window). As part of the source-localization process detailed below, data were re-referenced to a common average reference. This reference scheme is recommended for source-space investigations and used in prior whole-brain source-space connectome work (Wirsich et al., 2020, 2021). A monitored automated pipeline detected four kinds of signal anomalies: disconnected channels/flat signals, interelectrode electrolyte bridging (Tenke & Kayser, 2001), large amplitude deviations, and muscle/cap shift (motion) noise. Descriptives (e.g., temporal variance) were calculated for each electrode and 1-s time range. Epochs in which the signal exceeded four normalized median absolute deviations from the median (Rousseeuw & Croux, 1993) in 25% of the 1-s time range were removed. Electrodes for which the signal exceeded this value, 75% of the total recording time, were likewise removed. Among others, this approach is effective in removing periods with head motion artifacts. Ocular correction was performed using independent components (IC) analysis (infomax algorithm; Bell & Sejnowski, 1995) and joint consideration of temporal and spatial signal characteristics. The IC time series and inverse weights were compared with the time courses of the bipolar vertical or horizontal Electrooculography (EOG) and the inverse weight of a stereotypical blink or horizontal saccade to correct for vertical and horizontal ocular artifacts, respectively. If the squared joint temporal and spatial correlations for an IC exceeded an empirically calculated threshold based on Expectation Maximization (Mognon, Jovicich, Bruzzone, & Buiatti, 2011), that IC was subtracted from the data.

For source localization, we imported preprocessed EEG recordings and MR-based anatomical images into the Brainstorm software (Tadel, Baillet, Mosher, Pantazis, & Leahy, 2011). The EEG signals were resampled to 250 Hz, corrected for DC offsets, linearly detrended, and low-pass filtered at 70 Hz. We manually marked the fiducial points, including the anterior commissure, posterior commissure, interhemispheric point, nasion, and left and right preauricular points of all subjects using their individual anatomical images to aid the coregistration of electrode positions and T1 images. The coregistration was refined by manually moving the electrode positions onto the electrode artifacts visible in the T1 image. We then used the OpenMEEG software (Gramfort, Papadopoulo, Olivi, & Clerc, 2010) with a symmetric boundary element method (BEM) to calculate a forward model of the skull based on the individual T1 image of each subject (Tadel et al., 2019). Then, we used the Tikhonov-regularized minimum-norm estimation (MNE) inverse method to compute the sources, with default parameter settings for regularization and source depth weighting (Tikhonov parameter = 10%, assumed Signal-to-Noise Ratio (SNR) = 3.0, constrained sources normal to cortex, depth weighting 0.5/max amount 10; Baillet, Mosher, & Leahy, 2001; Tadel et al., 2019).

### Parcellation and Source-Leakage Correction

We used the Desikan-Killiany Atlas (Desikan et al., 2006) in Brainstorm to average source signals within each of the atlas’ 68 anatomically distinct brain regions. To aid with network-level interpretation, we also determined each region’s membership within the seven canonical intrinsic connectivity networks or ICNs (Yeo et al., 2011) based on spatial overlap. ICNs are commonly observed sets of distributed brain regions whose activity co-fluctuates in a correlated manner. The seven common networks we used comprise the default mode, frontoparietal, dorsal attention, salience/cingulo-opercular (or ventral attention), sensory-motor, visual, and limbic networks.

To mitigate source-leakage confounds caused by the blurring of point dipole sources and the spreading of signals across neighboring regions, we excluded regions whose signals were collinear with others based on a QR decomposition, which is commonly used to model the correlation structure of a set of variables, using the *qr* function in MATLAB. After examining the brain regions with highly collinear signals, we identified that the top 14 regions were limbic regions. As a result, 14 regions were excluded from the investigation due to the inherent challenges in capturing limbic signals with EEG (Supporting Information Figure S1). Specifically, limbic signals undergo significant attenuation as they traverse various brain tissues, making it challenging to differentiate limbic signals from those originating in the outer brain regions. The remaining 54 regional signals underwent detrending and bandpass filtering within canonical frequency ranges: delta (1–3 Hz), theta (4–7 Hz), alpha (8–12 Hz), beta (13–25 Hz), and gamma (30–45 Hz). Then, we used a symmetric orthogonalization procedure (Colclough, Brookes, Smith, & Woolrich, 2015) to remove all shared signal at zero lag between the regions. This multivariate method extends previous orthogonalization methods (Brookes, Woolrich, & Barnes, 2012; Hipp, Hawellek, Corbetta, Siegel, & Engel, 2012) and identifies orthogonal time courses that maintain the closest similarity to the original, unmodified time series. Finally, amplitude envelopes for each canonical frequency band and brain region were computed using the Hilbert transform, which were then downsampled to 40 Hz (Baker et al., 2014; Hunyadi, Woolrich, Quinn, Vidaurre, & De Vos, 2019). Such use of amplitude envelope aligns with the previous HMM studies on electrophysiological signals (MEG/EEG) (Baker et al., 2014; Coquelet et al., 2022; Quinn et al., 2018; Vidaurre et al., 2016), and the resulting band-limited amplitude coupling-based FC matrices, constituting connectome states, were found to be the most replicable measure for the source-localized resting-state MEG compared with other connectivity metrics (Colclough et al., 2016).

### HMM of Connectome States

**Figure 2.**
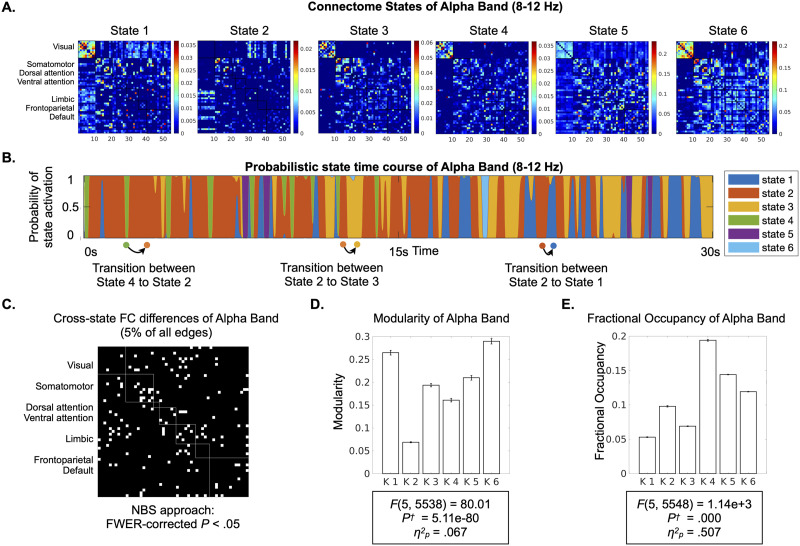
HMM states and state-dissociating features. (A) From band-specific leakage-corrected EEG signals (amplitude envelopes) concatenated over all subjects, HMM estimates connectome states that each has a characteristic FC matrix. The FC matrices reflect amplitude coupling among all region pairs. Here, the connectome states’ FC matrices for the alpha band (8–12 Hz) are provided as examples (see the full visualization of the states for all canonical bands in Supporting Information Figure S4). The rows and columns represent 54 regions organized according to their membership to canonical ICNs (listed on the left) (Yeo et al., 2011). (B) HMM estimates a specific (probabilistic) state time course for each subject, indicating when each state is active. An approximately 2-min section of the state time course is visualized for one subject, exemplifying periods occupied by each state and the transitions across states. (C) The binary matrix shows data-driven clusters of connections whose FC strength differed significantly across the six states. Specifically, *F* values from a connection-wise ANCOVA of the factor state were threshold at 5% connection density and entered NBS to control for multiple comparisons (Zalesky et al., 2010). For the ensuing significant cluster of connections, we provide the (D) Modularity and (E) Fractional Occupancy for each state. *F* values are reported for one-way ANCOVAs of the factor state (six levels) for each variable, adjusted for age and sex. Strong differences across states in all three measures suggest distinct spatial and temporal features of each state. *p*^†^, *p* values Bonferroni-corrected for 20 tests (four multivariate features and five frequency bands); *η*_*p*_^2^, partial eta-squared effect size.

The HMM assumes that time series data can be represented by a finite sequence of hidden states. Each HMM-inferred connectome state, along with its corresponding time series, represents a unique connectivity pattern that reoccurs over time. Using the HMM-MAR toolbox (Vidaurre et al., 2016), we applied the HMM to the region-wise EEG amplitude time series separately for each frequency band to derive discrete recurrent connectome states characterized by their mean activation and amplitude coupling-based FC matrix. We obtained six connectome states (*K* = 6; cf. Figures 1A and 2A for states’ FC matrices). While HMMs require an a priori selection of the number of states, *K*, the objective is not to establish a “correct” number of states but to strike a balance between model complexity and model fit and to identify a number that describes the dataset at a useful granularity (Quinn et al., 2018). Our previous fMRI-based investigation into connectome heritability (Jun et al., 2022) reported results for two different *K* values (to ensure that outcomes are not limited to a single chosen parameter), namely, *K* value of 4 and *K* value of 6. This choice was, in turn, informed by prior fMRI literatures (Karapanagiotidis et al., 2020; Vidaurre et al., 2016). The choice of *K* = 4 and *K* = 6 falls with the range applied in prior HMM studies of EEG and MEG data, which have used *K* values between 3 and 16 (Baker et al., 2014; Coquelet et al., 2022; Hunyadi et al., 2019; Quinn et al., 2018; Vidaurre et al., 2016), where two of the studies used a *K* value of 6. Therefore, based on the success of our prior fMRI study in revealing heritability within six-state and four-state models (Jun et al., 2022), the current study reports results from the *K* value of 6 (main text) and the *K* value of 4 (Supporting Information).

### Null Model of HMMs

To demonstrate that the dynamic trajectory of connectome state transitions is not occurring by chance, we employed a null model. This involved generating 50 simulated state time courses for each frequency band, which were of the same length as the original empirical state time courses. While preserving the static covariance structure, the temporal ordering of states was intentionally disrupted (Vidaurre et al., 2016). It is worth noting that selecting 50 simulations for each of the frequency bands in this analysis represents a rigorous choice in comparison with previous studies (e.g., four simulations in Vidaurre et al., 2017). We performed HMM inference with *K* values of 6 (and 4 for replication) on each of these simulated time courses, allowing us to recalculate all the above-described temporal and spatial connectome features at both the group and subject levels. Through this process, we confirmed that the original dataset’s nonrandom distribution of features over states represented veridical dynamics as it was absent in the simulated data (Supporting Information Figure S2). We further used the surrogate data for heritability testing as detailed below.

### Multivariate Temporal Features of the Dynamic Connectome

The HMM-derived estimates provide a comprehensive set of multivariate temporal features that simultaneously characterized all states of the dynamic connectome (Figure 1B, top). These estimates describe the temporal aspects of connectome dynamics by characterizing the sequence of connectome states, namely, the trajectory of the connectome through state space. For each subject, we calculated the Fractional Occupancy (the proportion of total time spent in a given state; 1 × *K*) and Transition Probability (the probability matrix of transitioning between all possible pairs of discrete states; *K* × *K*). Notably, our previous work demonstrated substantial genetic effects specifically on these two temporal features in fMRI-derived functional connectomes (Jun et al., 2022).

### Multivariate Spatial Features of the Dynamic Connectome

In line with our prior fMRI study (Jun et al., 2022), we also incorporated several multivariate spatial features to describe the FC arrangement of states (Figure 1B, bottom). While no spatial features were found to be heritable in the fMRI study, outcomes might differ for rapid EEG-derived dynamics. To assess the level of segregation for each connectome state, we estimated Newman’s Modularity (Newman, 2006), a fundamental global topological characteristic. The Brain Connectivity Toolbox (Rubinov & Sporns, 2010) was employed to quantify Modularity, where the modular partition was configured to comprise the canonical ICNs (Yeo et al., 2011). The Modularity value for the *K* states was then combined into a *K*-dimensional vector constituting the multivariate feature (Modularity_Time-Varying_) for heritability analysis.

Our second spatial feature was derived from clusters of connections that exhibited significant differences in FC strength across the *K* connectome states. As an initial step, we conducted mass univariate *F* tests across states for all connections, adjusting for age and sex. For each connection, the resulting (absolute) *F* value reflects its change in connectivity value across the *K* states. Subsequently, upon thresholding *F* values at a variety of connection densities (1%–5%), we performed the network-based statistics (NBS) permutation method to identify sets of connected edges or clusters that showed significant differences at family-wise error rate corrected *p* < 0.05 (see Supporting Information Figure S6 for visualization of the binary matrix of a data-driven set of clusters of connections). At each density, the edge-wise FC values within the identified clusters were averaged separately for each of the *K* states, resulting in a 1 × *K* vector representing FC_Time-Varying_ of the data-driven clusters for heritability analysis. In the main manuscript, we present results for 5% density and provide generalizations to other densities in Supporting Information Tables S3.

For exploratory analyses, we expanded our scope by incorporating a more comprehensive collection of multivariate spatial features. Specifically, we examined FC_Time-Varying_ between all pairs of the seven canonical ICNs, including the within-network connectivity of each ICN. For each ICN pair and each of the *K* states, we averaged FC values among their connections and combined them into a *K*-dimensional vector. This vector represents the multivariate feature that encompasses the FC_Time-Varying_ of the corresponding ICN pair for the purpose of heritability analysis (see Supporting Information Table S4 for details).

### Similarity Estimation and Heritability Testing

The procedures carried out in our previous twin heritability study (Jun et al., 2022) laid the groundwork for the current investigation (Figure 1C, top). Initially, for each multivariate connectome dynamics feature and separately for each frequency band, we constructed a multidimensional space by setting the origin point as the average of the feature from the 50 surrogate datasets, as described in the Null Model of HMMs section above. Using the multidimensional space, we calculated the pairwise similarity of each feature by measuring the Euclidean distance between pairs of subjects (Colclough et al., 2017; Jun et al., 2022). Crucially, this similarity estimation approach preserved the positional relationship between elements in each multivariate feature. To investigate the relationship between the genetic makeup of subject pairs and the similarity of each multivariate feature, we conducted a one-way analysis of covariance (ANCOVA) of sibling status (MZ twins, sex-matched DZ twins, and sex-matched unrelated individuals) on the similarity of each multivariate feature, adjusting for the difference in age and sex between the subject pairs.

Additionally, we explored whether the heritability of attributes was driven by the overall pattern or by specific components (i.e., state-by-state elements) of each multivariate feature. Specifically, we assessed the similarity of each state-specific component of the Fractional Occupancy, FC_Time-Varying_ of data-driven clusters, and Modularity_Time-Varying_ between a given pair of subjects. Likewise, we estimated the similarity of each off-diagonal state-pair component of the Transition Probability matrix. To examine the effects of sibling status and connectome state on the similarity of individual components of the multivariate features, we employed two-way ANCOVAs of the factors sibling status and connectome state.

Finally, we employed structural equation modeling, commonly used in classical twin studies, to quantify the variance in dynamic connectome features explained by genetic effects (Figure 1C, bottom). This modeling approach partitions the phenotypic variance into three distinct components using maximum likelihood methods: additive genetic variance (A), accompanied by either common environmental variance (C) or dominant genetic variance (D), and random environmental variance (E) (Yashin & Iachine, 1995). Leveraging these components, heritability is computed as the portion of phenotype variance explained by genetic variance, denoted as narrow-sense heritability (*h*^2^) in the ACE model and broad-sense heritability (*H*^2^) in the ADE model. Note that the classical twin design is grounded in several biological assumptions, which are necessary simplifications but are critical to keep in mind when interpreting twin study results on genetic and environmental influences. These include (a) the equal environments assumption, which posits that twins, regardless of zygosity, share similar environments; (b) the absence of gene–environment interaction, indicating consistent genetic effects across environments; and (c) the assumption of additive genetic effects, where the combined effects of individual genes determine the phenotype without considering dominance or epistasis (Keller & Coventry, 2005).

The genetic variance model requires each subject to have a singular value for each phenotype to estimate the correlation of univariate phenotypes among twin pairs. To accommodate our multivariate phenotypes within this framework, we computed the Euclidean distance for each subject’s multivariate phenotype from the origin point, which we established (Figure 1). To implement this method, we utilized the R package *mets* (https://cran.r-project.org/web/packages/mets/index.html), adjusted for age and sex. Subsequently, we employed nested models, namely, AE, CE, or DE, to gauge the statistical significance of these nested structures using a likelihood ratio test and assessed the fitness of each model using the Akaike information criterion (AIC) (Akaike, 1987).

## RESULTS

Results generated by the analysis procedures (illustrated in Figure 1) are presented following the progression from Figure 1A to 1C.

### Discrete Connectome States Have Distinct Spatial and Temporal Profiles

As a first step to quantifying dynamic connectome features, we identified discrete connectome states, that is, whole-brain recurrent connectivity patterns (Figure 2A), then showed that the states differ from each other in FC strength of specific networks, topological segregation-integration (Modularity), and Fractional Occupancy. To identify the states, we employed a data-driven approach using HMM (Vidaurre et al., 2016). Figure 2A visualizes states’ FC matrices, which reflect the spatial pattern of amplitude coupling. To ensure that results are not limited to the specific a priori selected number of states (*K*), we applied two different *K* values within the range of prior EEG/MEG studies (Baker et al., 2014; Coquelet et al., 2022; Hunyadi et al., 2019; Quinn et al., 2018; Vidaurre et al., 2016). We report outcomes for *K* = 6 and replicate results for *K* = 4 in the Supporting Information.

As expected, the states identified by the HMM switched rapidly (cf. Figure 2B illustrating a 30-s example of the probability of activation for the six states). The dwell time spent in individual occurrences of states were in the subsecond range for all EEG frequencies (mean across the *K* states and all subjects for delta (414.78 ± 293.0 ms), theta (344.53 ± 329.70 ms), alpha (344.01 ± 417.73 ms), beta (230.67 ± 408.14 ms), and gamma (250.05 ± 736.48 ms)).

Differences in FC strength across connectome states were quantified with a method that accounts for multiple comparisons in graph space (NBS; Zalesky et al., 2010). Figure 2C illustrates the cluster of connections with cross-state FC differences (NBS Family-wise error rate (FWER)-corrected *p*^†^ < 0.05) in alpha band, consisting of 70 out of 1,431 (5%) connections. Similar observations were made for all other EEG frequency bands (Supporting Information Figure S6 for the data-driven set of clusters of connections with different densities). In addition to examining the NBS-derived cluster, we conducted extensive exploratory analyses to assess the involvement of all canonical ICNs. We performed an equivalent one-way ANCOVA of the factor state on FC between each possible pair of canonical ICNs. The results demonstrated that all pairs of ICNs contributed to FC differences across states (Supporting Information Table S1 for *K* values of 6 and 4 across all frequency bands).

Moving beyond the specific sets of connections, we investigated cross-state differences in the global topology of the connectome, specifically focusing on Modularity (Newman, 2006; Rubinov & Sporns, 2010) due to its functional relevance (Sadaghiani et al., 2015; Shine & Poldrack, 2018). A one-way ANCOVA of the factor state showed significant difference in Modularity across the connectome states in the alpha band (Figure 2D; *F*_(5, 538)_ = 80.01, *p*^†^ = 5.11e−80, *η*_*p*_^2^ = 0.067). Similarly, this effect was present in all other EEG frequency bands (Supporting Information Table S2 for *K* values of 6 and 4 across all frequency bands).

Beyond the spatial features, the connectome states showed distinctive temporal characteristics. An equivalent one-way ANCOVA of the factor state on Fractional Occupancy revealed significant differences in the proportion of time spent in each state for the alpha band (Figure 2E; *F*_(5, 5548)_ = 1.14e+03, *p*^†^ < 0.001, where *p*^†^ is the *p* value Bonferroni-corrected for 20 tests, *η*_*p*_^2^ = 0.507). Similar observations were made for all other EEG frequency bands (Supporting Information Table S2 for *K* values of 6 and 4 across all frequency bands).

In summary, these findings confirm that the rapid (subsecond) dynamics of spontaneous connectivity can be characterized as nonrandom sequences of discrete connectome states, exhibiting differences in spatial organization, global topology, and proportion of occurrence.

### Multivariate Temporal Features of the Dynamic Connectome are Heritable

We tested the hypothesis that subject pairs with similar genetic makeup had more similar multivariate connectome dynamics features than subjects with less genetic relatedness. Specifically, the multivariate features included Fractional Occupancy (1 × *K*), Transition Probability matrix (*K* × *K*), FC_Time-Varying_ of data-driven clusters (1 × *K*), and Modularity_Time-Varying_ (1 × *K*; cf. Figure 1B). The similarity of each multivariate feature between a given pair of subjects was quantified as Euclidean distance (Figure 1C, top). Distance values entered a one-way ANCOVA of the factor sibling status with three levels, including MZ twins, sex-matched DZ twins, and sex-matched pairs of unrelated individuals, adjusted for age and sex. No subjects overlapped between groups.

**Figure 3.**
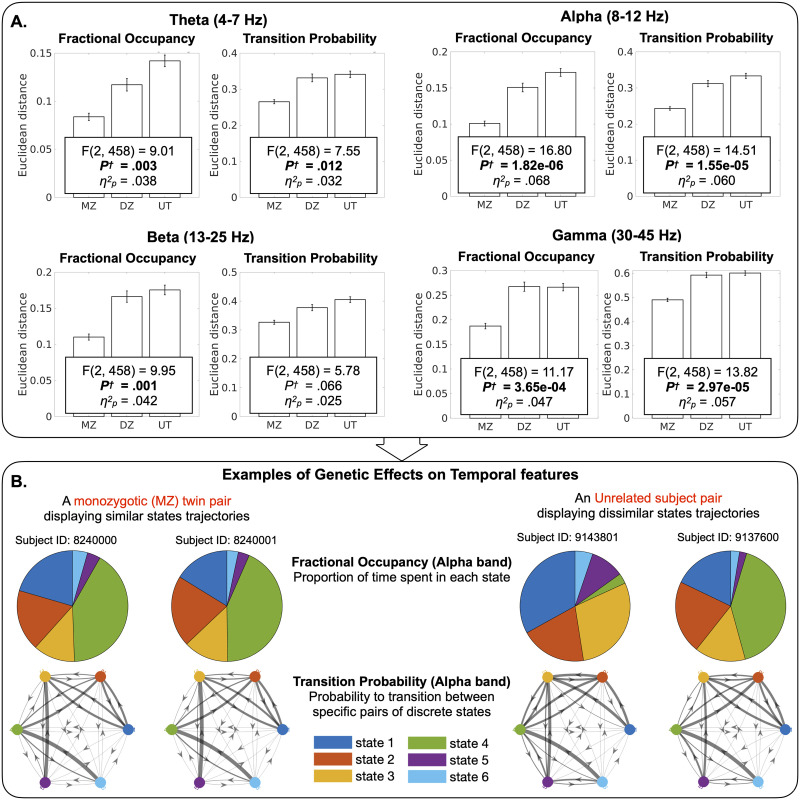
Heritability of temporal features of the dynamic connectome. (A) The heritability of each of the multivariate connectome dynamics features was assessed separately by one-way ANCOVAs of the factor sibling status (three levels: MZ twins, sex-matched DZ twins, and sex-matched pairs of unrelated individuals (UT)), adjusted for age and sex. The main effect of sibling status indicates the heritability, or genetic effect. Bar graphs show that genetically more similar subject pairs have shorter Euclidean distances, indicating the higher similarity of a given temporal feature. This effect of sibling status was large and independent of the chosen number of states (see Supporting Information Figure S5). *p*^†^: *p* values Bonferroni-corrected for 20 tests (four multivariate features and five frequency bands); *η*_*p*_^2^: Partial eta-squared effect size. (B) A visual illustration of the effects exemplified for two subject pairs, one from the MZ group (left) and the other from the unrelated subject group (right). While the MZ twins display high similarity of Fractional Occupancy and Transition Probabilities, both temporal features are dissimilar across the unrelated pair.

We found that temporal features describing the dynamic trajectory of connectome state transitions are heritable in the theta, alpha, beta, and gamma bands, but not in the delta band (Figure 3), that is, genetically closer subject pairs have more similar Fractional Occupancy and Transition Probability phenotypes compared with less genetically related pairs: Fractional Occupancy in theta (*F*_(2, 458)_ = 9.01, *p*^†^ = 0.003), alpha (*F*_(2, 458)_ = 16.80, *p*^†^ = 1.82e−06), beta (*F*_(2, 458)_ = 9.95, *p*^†^ = 0.001), and gamma (*F*_(2, 458)_ = 11.17, *p*^†^ = 3.65e−04) and Transition Probability in theta (*F*_(2, 458)_ = 7.55, *p*^†^ = 0.012), alpha (*F*_(2, 458)_ = 14.51, *p*^†^ = 1.55e−05), and gamma (*F*_(2, 458)_ = , *p*^†^ = 2.97e−05). This impact of sibling status on temporal features was consistently large and independent of the chosen number of states (cf. Figure 3 and Supporting Information Figure S5). Note that there was no effect of sibling status in the surrogate data lacking time-varying dynamics but with the preserved static covariance structure (cf. the Null Model of HMM section): Fractional Occupancy in delta through gamma (*F*_(2, 458)_ = 2.99, *F*_(2, 458)_ = 2.12, *F*_(2, 458)_ = 0.55, *F*_(2, 458)_ = 0.06, and *F*_(2, 458)_ = 0.07, respectively; all *p*^†^ = 1.00) and Transition Probability in delta through gamma (*F*_(2, 458)_ = 2.89, *F*_(2, 458)_ = 0.39, *F*_(2, 458)_ = 0.38, *F*_(2, 458)_ = 1.08, and *F*_(2, 458)_ = 0.54; all *p*^†^ = 1.00).

Contrary to the *temporal* features, we did not find support that was robust (i.e., invariant to methodological choices) for heritability of the *spatial* features, describing how connectome states are spatially instantiated in individuals. Specifically, outcomes of equivalent ANCOVAs for Modularity_Time-Varying_ and FC_Time-Varying_ of data-driven clusters showed no impact of sibling status, irrespective of frequency bands and the chosen number of states (Supporting Information Table S3). This lack of robust heritability was confirmed by a subsequent variance-component genetic analysis (see the Results section). Our sample size permitted detecting, at 80% power, effects of small size (*h*^2^ = 0.021, equivalent to *f* = 0.145, or larger). Therefore, if, for spatial features, heritability produced effects smaller than the detectable size, these effects would be of low practical impact. Further, we provide additional Bayesian factor values to directly assess the probability of H_0_ (i.e., the null hypothesis that there is no effect of sibling status) against H_1_ (Supporting Information Table S3). Indeed, the Bayes factor for Modularity_Time-Varying_ and FC_Time-Varying_ of data-driven clusters showed that the data are more likely to occur under H_0_ than under H_1_. For example, Modularity_Time-Varying_ in the alpha band showed anecdotal support for the null hypothesis (BF_01_ = 1.72), whereas FC_Time-Varying_ of data-driven clusters in the alpha band presented strong support for the null hypothesis (BF_01_ = 33.57), suggesting that data are 34 times more likely to occur under H_0_ than under H_1_. Notably, the lack of evidence for the heritability of spatial connectome dynamics features aligns with an equivalent null result in our preceding fMRI study (Jun et al., 2022).

To ensure that the absence of a robust outcome for spatial features was not influenced by a narrow feature selection, we performed additional equivalent analyses on exploratory spatial features: FC_Time-Varying_ of data-driven clusters defined across four other connection densities (1% ∼ 4% across different number of states; Supporting Information Table S3) and FC_Time-Varying_ of all 21 possible pairs among the seven ICNs (Supporting Information Tables S4). Consistent with our findings for the main spatial features, ANCOVAs of the factor sibling status and Bayes factors of all of the exploratory spatial features provided anecdotal to decisive evidence for H_0_, i.e., the lack of heritability. These findings strongly contrast the observations for temporal features, where the Bayes factor of both Fractional Occupancy and Transition Probability showed that the data are significantly more likely to occur under H_1_ than under H_0_: Fractional Occupancy in theta (BF_10_ = 1.07e+02), alpha (BF_10_ = 1.77e+05), beta (BF_10_ = 2.99e+02), and gamma (BF_10_ = 9.51e+02) and Transition Probability in theta (BF_10_ = 26.5), alpha (BF_10_ = 2.08e+04), beta (BF_10_ = 5.13), and gamma (BF_10_ = 9.72e+03).

Importantly, the effect sizes of significant multivariate Fractional Occupancy and Transition Probability were notably larger (with a mean *η*_*p*_^2^ of 0.049 across all frequency bands; Figure 3) compared with those of the *individual* (i.e., state-by-state) components of the multivariate features (with a mean *η*_*p*_^2^ of 0.016 across all frequency bands; Supporting Information Table S5). Therefore, our findings demonstrate that the dynamic trajectory of rapid EEG connectome state transitions are robustly heritable, predominantly when considered as multivariate patterns, rather than as individual state-specific components.

### Genetic Effects Account for Substantial Variability in Temporal Connectome Dynamics

We then quantified the extent to which genetic variance contributes to phenotypic variance using structural equation modeling, a technique commonly employed in classical twin studies (Falconer, 1990; Rijsdijk & Sham, 2002). While the model is traditionally applied to univariate phenotypes, the above-described one-way ANCOVAs of the factor sibling status suggest that connectome dynamics features are inherited predominantly as *multivariate* patterns. Therefore, we adapted the model to accommodate multivariate phenotypes by quantifying the subject-wise Euclidean distance of multivariate features from a “null” point of origin (from dynamics-free surrogate data; Figure 1C, bottom and the Null Model of HMM section).

A substantial portion of phenotypic variance in the temporal features was explained by genetic variance in the genetic models, adjusted for age and sex (Table 1; Supporting Information Table S6 for *K* = 4). Specifically, Transition Probability in alpha, beta, and gamma bands, as well as Fractional Occupancy in beta and gamma bands, were explained by the ADE model. In the ADE model, A (additive genetic effect) and D (dominant genetic effect) together estimate broad-sense heritability (*H*^2^). Conversely, Transition Probability in the theta band was described by the ACE model, where narrow-sense heritability (*h*^2^) was estimated using the A variance component. Notably, we found the substantial heritability of Transition Probability (*h*^2^ of the theta band = 38.4% [0.03, 0.74], *H*^2^ of the alpha band = 63.3% [0.56, 0.71], *H*^2^ of the beta band = 22.6% [0.08, 0.37], and *H*^2^ of the gamma band = 40% [0.28, 0.52]) and Fractional Occupancy (*H*^2^ of the beta band = 44.3% [0.33, 0.55] and *H*^2^ of the gamma band = 39.8% [0.28, 0.52]). In all cases, the fitness of the nested models (i.e., AE, CE, or DE) was not significantly better than the ACE or ADE model. These outcomes indicate that genetics contribute substantially to the temporal features of connectome dynamics.

**Table 1.** Variance-component model parameter estimates of the dynamic connectome features (*K* = 6)

Phenotypes	Genetic model	*h* ^2^	(95% CI)	A	(95% CI)	C/D	(95% CI)	E	(95% CI)	−2LL	AIC	Chi	Δ*df*	*p*(chi)
Delta														
Fractional Occupancy	ACE	0	(0.00, 0.00)	0.00	(0.00, 0.00)	0.04	(0.07, 0.15)	0.96	(0.85, 1.07)	−2,249.3	−2,237.3			
	AE	0.034	(−0.09, 0.16)	0.03	(0.09, 0.16)			0.97	(0.84, 1.09)	−2,249.1	−2,239.1	0.2	1	0.635
	CE	0	(0.00, 0.00)			0.04	(0.07, 0.15)	0.96	(0.85, 1.07)	−2,249.3	−2,239.3	0.2	1	0.635
Transition Probability	ACE	0.098	(−0.29, 0.48)	0.10	(0.29, 0.48)	0.11	(0.20, 0.43)	0.79	(0.65, 0.93)	−2,149.1	−2,137.1			
	AE	0.227	(0.10, 0.35)	0.23	(0.10, 0.35)			0.77	(0.65, 0.90)	−2,148.7	−2,138.7	0.4	1	0.528
	CE	0	(0.00, 0.00)			0.19	(0.08, 0.29)	0.81	(0.71, 0.92)	−2,148.9	−2,138.9	0.4	1	0.528
Modularity_Time-Varying_	ACE	0	(0.00, 0.00)	0.00	(0.00, 0.00)	0.04	(0.07, 0.14)	0.97	(0.86, 1.07)	459.8	471.8			
	AE	0.034	(−0.09, 0.16)	0.03	(0.09, 0.16)			0.97	(0.84, 1.09)	459.9	469.9	0.1	1	0.747
	CE	0	(0.00, 0.00)			0.04	(0.07, 0.14)	0.97	(0.86, 1.07)	459.8	469.8	0.1	1	0.747
FC_Time-Varying_ of data-driven clusters (5%)	ADE	0.094	(−0.07, 0.26)	0.00	(0.00, 0.00)	0.09	(0.07, 0.26)	0.91	(0.74, 1.07)	−6,711.4	−6,699.4			
	AE	0.092	(−0.00, 0.19)	0.09	(0.00, 0.19)			0.91	(0.81, 1.00)	−6,711.4	−6,701.4	0.1	1	0.782
	DE	0.094	(−0.00, 0.19)			0.09	(0.00, 0.19)	0.91	(0.81, 1.00)	−6,711.4	−6,701.4	0.1	1	0.782
Theta														
Fractional Occupancy	ACE	0	(0.00, 0.00)	0.00	(0.00, 0.00)	0.04	(0.07, 0.14)	0.96	(0.86, 1.07)	−2,199.3	−2,187.3			
	AE	0.025	(−0.10, 0.14)	0.03	(0.10, 0.14)			0.98	(0.86, 1.10)	−2,199.0	−2,189.0	0.3	1	0.604
	CE	0	(0.00, 0.00)			0.04	(0.07, 0.14)	0.96	(0.86, 1.07)	−2,199.3	−2,189.3	0.3	1	0.604
Transition Probability	ACE	0.384	(0.03, 0.74)	0.38	(0.03, 0.74)	0.07	(0.23, 0.38)	0.54	(0.44, 0.65)	−2,126.5	−2,114.5			
	AE	0.462	(0.36, 0.57)	0.46	(0.36, 0.57)			0.54	(0.43, 0.64)	−2,126.3	−2,116.3	0.2	1	0.666
	CE	0	(0.00, 0.00)			0.38	(0.28, 0.47)	0.63	(0.53, 0.72)	−2,122.1	−2,112.1	0.2	1	0.666
Modularity_Time-Varying_	ACE	0	(−0.00, 0.00)	0.00	(0.00, 0.00)	0.06	(0.05, 0.17)	0.94	(0.83, 1.05)	448.1	46.1			
	AE	0.071	(−0.06, 0.20)	0.07	(0.06, 0.20)			0.93	(0.80, 1.06)	448.3	458.3	0.1	1	0.716
	CE	0	(0.00, 0.00)			0.06	(0.05, 0.17)	0.94	(0.83, 1.05)	448.1	458.1	0.1	1	0.716
FC_Time-Varying_ of data-driven clusters (5%)	ACE	0	(0.00, 0.00)	0.00	(0.00, 0.00)	0.11	(0.01, 0.20)	0.89	(0.80, 0.99)	−6,411.4	−6,399.4			
	AE	0.101	(0.00, 0.20)	0.10	(0.00, 0.20)			0.90	(0.80, 1.00)	−6,411.0	−6,401.0	0.4	1	0.546
	CE	0	(0.00, 0.00)			0.11	(0.01, 0.20)	0.89	(0.80, 0.99)	−6,411.4	−6,401.4	0.4	1	0.546
Alpha														
Fractional Occupancy	ACE	0	(−0.00, 0.00)	0.00	(0.00, 0.00)	0.05	(0.06, 0.16)	0.95	(0.84, 1.06)	−2,313.0	−24.0			
	AE	0.057	(−0.07, 0.18)	0.06	(0.07, 0.18)			0.94	(0.82, 1.07)	−2,312.8	−2,302.8	0.1	1	0.704
	CE	0	(0.00, 0.00)			0.05	(0.06, 0.16)	0.95	(0.84, 1.06)	−2,313.0	−2,303.0	0.1	1	0.704
Transition Probability	ADE	0.633	(0.56, 0.71)	0.32	(0.32, 0.96)	0.31	(0.33, 0.96)	0.37	(0.29, 0.44)	−1,787.0	−1,775.0			
	AE	0.627	(0.55, 0.71)	0.63	(0.55, 0.71)			0.37	(0.29, 0.45)	−1,786.1	−1,776.1	0.8	1	0.364
	DE	0.635	(0.56, 0.71)			0.64	(0.56, 0.71)	0.37	(0.29, 0.44)	−1,786.2	−1,776.2	0.8	1	0.364
Modularity_Time-Varying_	ACE	0.01	(−0.43, 0.45)	0.01	(0.43, 0.45)	0.07	(0.32, 0.45)	0.93	(0.79, 1.06)	631.7	643.7			
	AE	0.082	(−0.04, 0.21)	0.08	(0.04, 0.21)			0.92	(0.79, 1.04)	631.8	641.8	0.1	1	0.752
	CE	0	(0.00, 0.00)			0.07	(0.04, 0.18)	0.93	(0.82, 1.04)	631.7	641.7	0.1	1	0.752
FC_Time-Varying_ of data-driven clusters (5%)	ADE	0.058	(−0.04, 0.15)	0.06	(0.04, 0.15)	0.00	(0.00, 0.00)	0.94	(0.85, 1.04)	−6,214.0	−6,202.0			
	AE	0.058	(−0.04, 0.15)	0.06	(0.04, 0.15)			0.94	(0.85, 1.04)	−6,214.0	−6,204.0	1.6	1	1
	DE	0.058	(−0.04, 0.15)			0.06	(0.04, 0.15)	0.94	(0.85, 1.04)	−6,214.0	−6,204.0	1.6	1	1
Beta														
Fractional Occupancy	ADE	0.443	(0.33, 0.55)	0.04	(0.49, 0.57)	0.40	(0.15, 0.96)	0.56	(0.45, 0.67)	−2,468.4	−2,456.4			
	AE	0.425	(0.32, 0.53)	0.43	(0.32, 0.53)			0.58	(0.47, 0.68)	−2,467.1	−2,457.1	1.3	1	0.263
	DE	0.444	(0.34, 0.55)			0.44	(0.34, 0.55)	0.56	(0.45, 0.66)	−2,468.4	−2,458.4	1.3	1	0.263
Transition Probability	ADE	0.226	(0.08, 0.37)	0.00	(0.00, 0.00)	0.23	(0.08, 0.37)	0.77	(0.63, 0.92)	−2,223.8	−2,211.8			
	AE	0.192	(0.06, 0.33)	0.19	(0.06, 0.33)			0.81	(0.67, 0.94)	−2,222.5	−2,212.5	1.3	1	0.251
	DE	0.226	(0.08, 0.37)			0.23	(0.08, 0.37)	0.77	(0.63, 0.92)	−2,223.8	−2,213.8	1.3	1	0.251
Modularity_Time-Varying_	ADE	0.118	(−0.01, 0.25)	0.00	(0.00, 0.00)	0.12	(0.01, 0.25)	0.88	(0.75, 1.01)	614.3	626.3			
	AE	0.105	(−0.02, 0.23)	0.11	(0.02, 0.23)			0.90	(0.77, 1.02)	614.8	624.8	0.5	1	0.477
	DE	0.118	(−0.01, 0.25)			0.12	(0.01, 0.25)	0.88	(0.75, 1.01)	614.3	624.3	0.5	1	0.477
FC_Time-Varying_ of data-driven clusters (5%)	ACE	0	(0.00, 0.00)	0.00	(0.00, 0.00)	0.02	(0.07, 0.12)	0.98	(0.88, 1.07)	−5,061.8	−5,049.8			
	AE	0.02	(−0.09, 0.13)	0.02	(0.09, 0.13)			0.98	(0.87, 1.09)	−5,061.7	−5,051.7	0.1	1	0.792
	CE	0	(0.00, 0.00)			0.02	(0.07, 0.12)	0.98	(0.88, 1.07)	−5,061.8	−5,051.8	0.1	1	0.792
Gamma														
Fractional Occupancy	ADE	0.398	(0.28, 0.52)	0.00	(0.00, 0.00)	0.40	(0.28, 0.52)	0.60	(0.48, 0.72)	−2,013.3	−2,001.3			
	AE	0.358	(0.24, 0.48)	0.36	(0.24, 0.48)			0.64	(0.52, 0.76)	−2,008.9	−1,998.9	4.4	1	0.035
	DE	0.398	(0.28, 0.52)			0.40	(0.28, 0.52)	0.60	(0.48, 0.72)	−2,013.3	−2,003.3	4.4	1	0.035
Transition Probability	ADE	0.4	(0.28, 0.52)	0.00	(0.00, 0.00)	0.40	(0.28, 0.52)	0.60	(0.48, 0.72)	−1,372.4	−136.4			
	AE	0.376	(0.26, 0.49)	0.38	(0.26, 0.49)			0.62	(0.51, 0.74)	−137.9	−136.9	1.5	1	0.222
	DE	0.4	(0.28, 0.52)			0.40	(0.28, 0.52)	0.60	(0.48, 0.72)	−1,372.4	−1,362.4	1.5	1	0.222
Modularity_Time-Varying_	ADE	0	(0.00, 0.00)	0.00	(0.00, 0.00)	0.00	(0.00, 0.00)	1.00	(1.00, 1.00)	695.2	707.2			
	AE	0	(0.00, 0.00)	0.00	(0.00, 0.00)			1.00	(1.00, 1.00)	695.2	705.2	4.4	1	1
	DE	0	(−0.00, 0.00)			0.00	(0.00, 0.00)	1.00	(1.00, 1.00)	695.2	705.2	4.4	1	1
FC_Time-Varying_ of data-driven clusters (5%)	ACE	0	(0.00, 0.00)	0.00	(0.00, 0.00)	0.00	(0.00, 0.00)	1.00	(1.00, 1.00)	−6,176.5	−6,164.5			
	AE	0	(0.00, 0.00)	0.00	(0.00, 0.00)			1.00	(1.00, 1.00)	−6,176.5	−6,166.5	2.8	1	1
	CE	0	(0.00, 0.00)			0.00	(0.00, 0.00)	1.00	(1.00, 1.00)	−6,176.5	−6,166.5	2.8	1	1

All models were adjusted for age and sex. The AE and CE models are nested within the ACE model, and AE and DE models are nested within the ADE model. Each nested model was compared with the full model. The boldfaced entries indicate the full models with nonzero heritability (*h*^2^ column) and confidence intervals not crossing 0 (95% CI column next to the *h*^2^ column). These models were chosen because the fitness of the nested models did not significantly improve compared with the full model (*p*(chi) column). The fitness of models was tested based on a change in AIC (for a change of *df* of 1, the statistically significant change in *χ*^2^ is 3.84). *h*^2^, the narrow-sense heritability was estimated as *σ*^2^_A_/(*σ*^2^_A_ + *σ*^2^_C_ + *σ*^2^_E_); the broad-sense heritability was estimated as (*σ*^2^_A_ + *σ*^2^_D_)/(*σ*^2^_A_ + *σ*^2^_D_ + *σ*^2^_E_). CI, confidence interval (lower bound, upper bound); A, additive genetic effect; C, common environmental effect; D, dominant genetic effect; E, unique/nonshared environment effect; −2LL, twice the negative log-likelihood; AIC, Akaike information criterion; *df*, degrees of freedom; *χ*^2^, chi square, Δ*df*, change in degree of freedom between the full model and the nested model; *p*, *χ*^2^ test in model fitting.

Consistent with the ANCOVA-based heritability findings (Supporting Information Tables S3–S4), the genetic models did not support genetic effects on either spatial features (Modularity_Time-Varying_ and FC_Time-Varying_ of data-driven clusters; Table 1 and Supporting Information Table S6 for *K* value of 4). Specifically, the heritability (*h*^2^) of spatial features was estimated as 0 or its 95% confidence interval crossed 0, supporting the null hypothesis, i.e., lack of heritability.

## DISCUSSION

The use of source-space electrophysiological signals allowed us to investigate rapidly emerging and dissolving patterns of spatially localized connectivity networks and their transitions at the whole-brain level. Bringing this approach to a large cohort, we established genetic effects on rapid connectome dynamics in specific frequency bands. Overcoming the limited temporal resolution of prior heritability investigations of slow connectome dynamics in fMRI (cf. infraslow (<0.1 Hz) fMRI BOLD signal; Barber, Hegarty, Lindquist, & Karlsgodt, 2021; Jun et al., 2022; Vidaurre et al., 2017), the current findings shed light on subsecond timescales highly relevant to cognitive processes. As an additional innovation, we embraced the multidimensional nature of dynamic connectome features (Jun et al., 2022), as these features collectively encompass patterns from multiple connectome states. Reinforcing the multidimensional view, the heritability effect size was larger for *multivariate* features (Figure 3 and Supporting Information Figure S5) than for state-wise scalar features (cf. Supporting Information Table S5). Quantitative modeling with the multivariate features showed that the genetic influence on the rapid sequencing of connectome states was substantial (22%–63% of variance explained).

Previous twin studies have found considerable genetic effects on multiple EEG features, however, predominantly focusing on stable rather than time-varying characteristics. Specifically, in terms of the power spectrum, heritability was particularly evident in individuals’ alpha peak frequency (71% to 83% phenotypic variance explained; Posthuma, Neale, Boomsma, & de Geus, 2001; C. M. Smit, Wright, Hansell, Geffen, & Martin, 2006) and alpha power (79% to 93% variance explained, depending on scalp location and age of cohort; C. M. Smit et al., 2006; D. J. A. Smit et al., 2005). The degree of heritability observed beyond the alpha band has been somewhat less pronounced; in direct comparisons across the spectrum, the highest heritability of spectral power was observed at the alpha peak frequency, while it was lower in the theta and delta bands (D. J. A. Smit et al., 2005).

Besides the EEG power spectrum, static (time-averaged) MEG/EEG FC measures were found to be subject-specific (cf. fingerprinting; Kabbara, Paban, & Hassan, 2021; Kong, Wang, Xu, Babiloni, & Chen, 2019; Sareen et al., 2021) and under genetic influence. For example, the heritability estimates of static EEG FC ranged from 27% to 75%, primarily observed in alpha and beta bands (Posthuma et al., 2005; Schutte et al., 2013). Additionally, heritability estimates for graph theoretical measures of the static EEG FC matrix ranged from 46% to 89% for clustering coefficients and from 37% to 62% for the average path length (D. J. A. Smit, Stam, Posthuma, Boomsma, & de Geus, 2008). However, it is important to note that the abovementioned studies were performed in sensor-space and employed synchronization likelihood for network estimation, a measure that may be susceptible to volume conduction artifacts. A *source-space* MEG heritability study correcting such artifacts estimated the heritability of amplitude coupling in individual edges, which averaged across edges and reached 8% for the alpha band and 19% for the beta band (Colclough et al., 2017).

Our study significantly extends this important previous research by establishing the heritability of rapid whole-brain connectome dynamics, derived from source-localized (and leakage-corrected) EEG. Our quantitative heritability estimates fall within the range reported in the above-described studies that focused on stable EEG spectral properties and static FC investigations. The strongest genetic influence in our study was observed in the alpha band, paralleling prior heritability estimates of the power spectrum. While this dominance may result from the high signal-to-noise ratio of the alpha band, the strong alpha signal in itself likely reflects an important functional role of this frequency in cognition (Klimesch, 2012; Palva & Palva, 2011; Sadaghiani & Kleinschmidt, 2016). In general, and for all frequency bands, it is the rapid changes that are thought to be particularly critical for cognitive processes, which are inherently dynamic at numerous timescales (Gratton, 2018). By addressing electrophysiological processes from a time-varying perspective, the current study establishes the heritability of cognitively significant rapid brain state changes. Our separate study in the same cohort directly confirms the predicted implications of such rapid connectome dynamics for individual differences in cognitive abilities (Jun et al., 2024).

Notably, robust evidence for a genetic influence was found for Transition Probabilities in all bands except delta but was observed for Fractional Occupancy in the higher bands only (beta and gamma). While Transition Probability and Fractional Occupancy are not fully independent measures, they contain nonoverlapping information about connectome dynamics. For example, a state with a particularly high Fractional Occupancy is likely to have high values as initial state and target state in the Transition Probability matrix. Despite such dependence, however, two hypothetical subjects with highly comparable Fractional Occupancy values across the *K* states may still have substantially different sequencing and, thus, transition probabilities across the states. This sequencing, as suggested by our findings, is under substantial genetic influence broadly across electrophysiological timescales. At least at infraslow timescales (typically observed in fMRI), such brain state changes are, in part, driven by the spatially broad but structured influence of ascending modulatory neurotransmitter systems (Klaassens et al., 2017; Lord et al., 2019; Shine, van den Brink, Hernaus, Nieuwenhuis, & Poldrack, 2018). Numerous genetic polymorphisms with functional impact are known within receptors, transporters, and enzymes of these systems (de Rojas et al., 2021; Kautzky et al., 2015; Sadaghiani et al., 2017). Future causal (e.g., neuropharmacological interventions and subcortical microstimulation) or modeling studies could assess to what degree similar neuromodulatory processes are at play in the individually specific connectome dynamics at rapid timescales.

Interestingly, alongside the substantial genetic influence (*h*^2^ ranging from 22.6% to 63.3%; Table 1), a considerable amount of unique environmental influence (E component ranging from 37% to 77%; Table 1) was consistently observed across the heritable phenotypes. Such notable impact of unique environmental factors suggests that differential parenting, individual life events, personal choices, and/or gene–environment interactions may contribute significantly to the sequencing and the duration of rapidly transitioning brain states, leading to differences between twins within the same family. Our findings align with previous heritability studies on *static* MEG/EEG FC measures, which have also reported a substantial impact of unique environmental factors (Posthuma et al., 2005; Schutte et al., 2013). A strong impact from a unique environment is in line with the fact that neural wiring, which builds the structural backbone for FC dynamics, is shaped not only by genetics but also under significant environmental influences. Examples include thalamocortical projection pathways being refined by neural activity (Catalano & Shatz, 1998), cortical synaptogenesis in human and rhesus infants that sets the foundation for the refinement of cognitive development by experience-dependent plasticity mechanisms (Goldman-Rakic, 1987; Vanderhaeghen & Polleux, 2023), programming of the prefrontal-limbic system in response to stress-related endocrine mediators (Tost, Champagne, & Meyer-Lindenberg, 2015), and hippocampal neurogenesis in human adults triggered by learning (Gould, Beylin, Tanapat, Reeves, & Shors, 1999). Note, however, that the E component also encompasses measurement error, which can introduce noise into the data, leading to an overestimation or underestimation of the true effect of unique environmental factors on the phenotypes.

Consistent with our previous fMRI investigation (Jun et al., 2022), the results concerning the spatial features of connectome states largely supported the null hypothesis of the absence of heritability for a wide range of features. Specifically, when examining our primary spatial features, i.e., cluster-based FC_Time-Varying_ and Modularity_Time-Varying_, there was no discernible influence of sibling status on the phenotypic similarity between subjects across different number of states. This lack of heritability for cluster-based FC_Time-Varying_ was further corroborated by exploring additional connection densities (Supporting Information Table S3). Further, in an exhaustive exploratory assessment of 21 ICN pairs, FC_Time-Varying_ exhibited no observable influence from the sibling status under different methodological alternatives (Supporting Information Table S4). Therefore, our study suggests that genetic effects primarily contribute to how the connectome *transitions* across different states, rather than the precise way in which the states are spatially instantiated in individuals. It is remarkable that this dissociation between temporal and spatial features of connectome dynamics holds across the full spectrum of connectivity timescales, from infraslow (fMRI) to the gamma band (EEG). Still, it is important to acknowledge that factors beyond genetics, such as individuals’ experiences and learning, play a substantial role in shaping subject-specific connectomes and their spatial patterns, as indicated by the significant contribution of common and random environmental variances to the spatial features in Table 1.

Our study is subject to several limitations and methodological considerations. While we provide results separately for each canonical frequency band, this approach does not assume or necessitate the bands to be discretely separable or oscillatory in nature. The approach is equally compatible with a more general view that the bands represent electrophysiological processes at different speeds within a larger 1/*f* spectrum. Further, we defined the boundaries of the frequency bands according to common conventions in the field rather than according to the individual’s power spectrum. Because the latter (especially the alpha peak frequency commonly used to anchor individual bands) is highly heritable (see above), defining the bands individually may strengthen the observed heritability of connectome dynamics. However, a nonindividualized definition of bands is unlikely to result in false positives in terms of such heritability. Another consideration is that while the set of spatial features of connectome dynamics in our main and supplementary reports was large, it is necessarily inexhaustive. Other dynamic spatial features not explored in this study could potentially exhibit heritability and call for future investigation.

In conclusion, our findings provide the first evidence of genetic influence on rapid transitions between whole-brain source-space EEG connectome states and the proportion of time spent in each state. In combination with our previous findings in fMRI-derived dynamics (Jun et al., 2022), the evidence of a genetic basis of connectome state trajectories extends the full breadth of connectivity timescales from infraslow to the gamma band. Extensive prior work has established that various aspects of brain anatomy are heritable, including how the brain is wired in terms of white matter tracts (Kochunov et al., 2015; Sha, Schijven, Fisher, & Francks, 2023). The genetic influence on wiring has further been extended to the time-averaged functional connectome (Colclough et al., 2017; Schutte et al., 2013; Sinclair et al., 2015), reflecting which brain regions have a strong (or respectively weak) average tendency to coordinate their activity. While this important prior research has focused on the brain’s stable structural/functional architecture, the current work shifted the focus to the brain’s dynamic behavior (i.e., what the brain *does* in terms of time-varying dynamics). Interestingly, the shift to time-varying connectome states showed that genetics impacts the *act of transitioning* across states rather than the spatial organization of those states (at least among the features we studied). This observation gains particular importance in light of the central role of rapid electrophysiological dynamics in brain function and cognition (Gratton, 2018). Our findings may inform the identification of functionally relevant genetic polymorphisms and the development of connectome-based biomarkers at timescales particularly relevant to cognitive processes.

## ACKNOWLEDGMENTS

We thank Dr. Andre Altmann for his extensive guidance in analytic approaches and Drs. Jonathan Wirsich and Thomas Alderson for their guidance in data preprocessing. Computational resources for this work were provided by the Minnesota Supercomputing Institute at the University of Minnesota Informatics Institute. The Center for Magnetic Resonance Research (supported by Grant Nos. NIBIB P41 EB027061 and 1S10OD017974-01) at the University of Minnesota provided resources that contributed to the MRI-related results reported within this article. The original data collection of the data analyzed in this paper was funded by NIH grants R37 DA05147 and R01 DA036216. This work was partly supported by the National Institute for Mental Health (1R01MH116226 to Sepideh Sadaghiani).

## SUPPORTING INFORMATION

Supporting information for this article is available at https://doi.org/10.1162/netn_a_00391.

## AUTHOR CONTRIBUTIONS

Suhnyoung Jun: Conceptualization; Formal analysis; Investigation; Methodology; Writing – original draft. Thomas H. Alderson: Methodology. Stephen Malone: Data curation; Funding acquisition; Project administration; Resources. Jeremy Harper: Methodology. Ruskin H. Hunt: Methodology. Kathleen M. Thomas: Methodology. William Iacono: Funding acquisition; Project administration. Sylia Wilson: Data curation. Sepideh Sadaghiani: Conceptualization; Funding acquisition; Methodology; Project administration; Supervision; Writing – original draft.

## FUNDING INFORMATION

Sepideh Sadaghiani, National Institute of Mental Health and Neurosciences (https://dx.doi.org/10.13039/100019274), Award ID: 1R01MH116226. The University of Minnesota Center for Magnetic Resonance Research, National Institute of Biomedical Imaging and Bioengineering (https://dx.doi.org/10.13039/100000070), Award ID: P41 EB027061. The University of Minnesota Center for Magnetic Resonance Research, National Institute of Biomedical Imaging and Bioengineering (https://dx.doi.org/10.13039/100000070), Award ID: P41 1S10OD017974-01. The University of Minnesota Center for Magnetic Resonance Research, National Institutes of Health (https://dx.doi.org/10.13039/100000002), Award ID: R37 DA05147. The University of Minnesota Center for Magnetic Resonance Research, National Institutes of Health (https://dx.doi.org/10.13039/100000002), Award ID: R01 DA036216.

## Supplementary Material



## TECHNICAL TERMS

Source-leakage: This phenomenon where the estimated sources of neural activity are affected by artifacts or signals that are not of interest.
Connectome states: Discrete functional brain connectivity patterns recurring over time.
Heritability: Proportion of phenotypic variation due to genetic factors.
Hidden Markov model (HMM): Statistical model for sequences with unobserved states.
Orthogonalization: Process used to isolate a specific brain activity from noise or other interfering signals.
Multivariate dynamic connectome phenotypes: Phenotypes that comprehensively represent the time-varying changes of all states.
Euclidean distance: Measure of the straight-line distance between two points in Euclidean space.
Variance component modeling: Statistical method to partition observed phenotypic variance into different sources, such as genetic and environmental, in a mixed-effects model.
Twin study design: Research design comparing similarities between monozygotic and dizygotic twins to study genetic and environmental influences.
Phenotypes: Observable traits influenced by genetics and environment.

## References

[bib1] Akaike, H. (1987). Factor analysis and AIC. Psychometrika, 52(3), 317–332. 10.1007/BF02294359

[bib2] Baillet, S., Mosher, J. C., & Leahy, R. M. (2001). Electromagnetic brain mapping. IEEE Signal Processing Magazine, 18(6), 14–30. 10.1109/79.962275

[bib3] Baker, A. P., Brookes, M. J., Rezek, I. A., Smith, S. M., Behrens, T., Probert Smith, P. J., & Woolrich, M. (2014). Fast transient networks in spontaneous human brain activity. eLife, 3, e01867. 10.7554/eLife.01867, 24668169 PMC3965210

[bib4] Barber, A. D., Hegarty, C. E., Lindquist, M., & Karlsgodt, K. H. (2021). Heritability of functional connectivity in resting state: Assessment of the dynamic mean, dynamic variance, and static connectivity across networks. Cerebral Cortex, 31(6), 2834–2844. 10.1093/cercor/bhaa391, 33429433 PMC8325018

[bib5] Bell, A. J., & Sejnowski, T. J. (1995). An information-maximization approach to blind separation and blind deconvolution. Neural Computation, 7(6), 1129–1159. 10.1162/neco.1995.7.6.1129, 7584893

[bib6] Brookes, M. J., O’Neill, G. C., Hall, E. L., Woolrich, M. W., Baker, A., Palazzo Corner, S., … Barnes, G. R. (2014). Measuring temporal, spectral and spatial changes in electrophysiological brain network connectivity. NeuroImage, 91, 282–299. 10.1016/j.neuroimage.2013.12.066, 24418505

[bib7] Brookes, M. J., Woolrich, M., Luckhoo, H., Price, D., Hale, J. R., Stephenson, M. C., … Morris, P. G. (2011). Investigating the electrophysiological basis of resting state networks using magnetoencephalography. Proceedings of the National Academy of Sciences, 108(40), 16783–16788. 10.1073/pnas.1112685108, 21930901 PMC3189080

[bib8] Brookes, M. J., Woolrich, M. W., & Barnes, G. R. (2012). Measuring functional connectivity in MEG: A multivariate approach insensitive to linear source leakage. NeuroImage, 63(2), 910–920. 10.1016/j.neuroimage.2012.03.048, 22484306 PMC3459100

[bib9] Catalano, S. M., & Shatz, C. J. (1998). Activity-dependent cortical target selection by thalamic axons. Science, 281(5376), 559–562. 10.1126/science.281.5376.559, 9677198

[bib10] Chorlian, D. B., Tang, Y., Rangaswamy, M., O’Connor, S., Rohrbaugh, J., Taylor, R., & Porjesz, B. (2007). Heritability of EEG coherence in a large sib-pair population. Biological Psychology, 75(3), 260–266. 10.1016/j.biopsycho.2007.03.006, 17498861 PMC2270612

[bib11] Cohen, J. R. (2018). The behavioral and cognitive relevance of time-varying, dynamic changes in functional connectivity. NeuroImage, 180, 515–525. 10.1016/j.neuroimage.2017.09.036, 28942061 PMC6056319

[bib12] Colclough, G. L., Brookes, M. J., Smith, S. M., & Woolrich, M. W. (2015). A symmetric multivariate leakage correction for MEG connectomes. NeuroImage, 117, 439–448. 10.1016/j.neuroimage.2015.03.071, 25862259 PMC4528074

[bib13] Colclough, G. L., Smith, S. M., Nichols, T. E., Winkler, A. M., Sotiropoulos, S. N., Glasser, M. F., … Woolrich, M. W. (2017). The heritability of multi-modal connectivity in human brain activity. eLife, 6, e20178. 10.7554/eLife.20178, 28745584 PMC5621837

[bib14] Colclough, G. L., Woolrich, M. W., Tewarie, P. K., Brookes, M. J., Quinn, A. J., & Smith, S. M. (2016). How reliable are MEG resting-state connectivity metrics? NeuroImage, 138, 284–293. 10.1016/j.neuroimage.2016.05.070, 27262239 PMC5056955

[bib15] Coquelet, N., De Tiège, X., Roshchupkina, L., Peigneux, P., Goldman, S., Woolrich, M., & Wens, V. (2022). Microstates and power envelope hidden Markov modeling probe bursting brain activity at different timescales. NeuroImage, 247, 118850. 10.1016/j.neuroimage.2021.118850, 34954027 PMC8803543

[bib16] Deligianni, F., Centeno, M., Carmichael, D. W., & Clayden, J. D. (2014). Relating resting-state fMRI and EEG whole-brain connectomes across frequency bands. Frontiers in Neuroscience, 8, 258. 10.3389/fnins.2014.00258, 25221467 PMC4148011

[bib17] Delorme, A., & Makeig, S. (2004). EEGLAB: An open source toolbox for analysis of single-trial EEG dynamics including independent component analysis. Journal of Neuroscience Methods, 134(1), 9–21. 10.1016/j.jneumeth.2003.10.009, 15102499

[bib18] de Pasquale, F., Della Penna, S., Snyder, A. Z., Lewis, C., Mantini, D., Marzetti, L., … Corbetta, M. (2010). Temporal dynamics of spontaneous MEG activity in brain networks. Proceedings of the National Academy of Sciences, 107(13), 6040–6045. 10.1073/pnas.0913863107, 20304792 PMC2851876

[bib19] de Rojas, I., Moreno-Grau, S., Tesi, N., Grenier-Boley, B., Andrade, V., Jansen, I. E., … Ruiz, A. (2021). Common variants in Alzheimer’s disease and risk stratification by polygenic risk scores. Nature Communications, 12(1), 3417. 10.1038/s41467-021-22491-8, 34099642 PMC8184987

[bib20] Desikan, R. S., Ségonne, F., Fischl, B., Quinn, B. T., Dickerson, B. C., Blacker, D., … Killiany, R. J. (2006). An automated labeling system for subdividing the human cerebral cortex on MRI scans into gyral based regions of interest. NeuroImage, 31(3), 968–980. 10.1016/j.neuroimage.2006.01.021, 16530430

[bib21] Douw, L., Wakeman, D. G., Tanaka, N., Liu, H., & Stufflebeam, S. M. (2016). State-dependent variability of dynamic functional connectivity between frontoparietal and default networks relates to cognitive flexibility. Neuroscience, 339, 12–21. 10.1016/j.neuroscience.2016.09.034, 27687802 PMC5635855

[bib22] Eichenbaum, A., Pappas, I., Lurie, D., Cohen, J. R., & D’Esposito, M. (2021). Differential contributions of static and time-varying functional connectivity to human behavior. Network Neuroscience, 5(1), 145–165. 10.1162/netn_a_00172, 33688610 PMC7935045

[bib23] Falconer, D. S. (1990). Introduction to quantitative genetics (3rd ed.). Longman Group.

[bib24] Goldman-Rakic, P. S. (1987). Development of cortical circuitry and cognitive function. Child Development, 58(3), 601–622. 10.2307/1130201, 3608641

[bib25] Gould, E., Beylin, A., Tanapat, P., Reeves, A., & Shors, T. J. (1999). Learning enhances adult neurogenesis in the hippocampal formation. Nature Neuroscience, 2(3), 260–265. 10.1038/6365, 10195219

[bib26] Gramfort, A., Papadopoulo, T., Olivi, E., & Clerc, M. (2010). OpenMEEG: Opensource software for quasistatic bioelectromagnetics. BioMedical Engineering OnLine, 9(1), 45. 10.1186/1475-925X-9-45, 20819204 PMC2949879

[bib27] Gratton, G. (2018). Brain reflections: A circuit-based framework for understanding information processing and cognitive control. Psychophysiology, 55(3), e13038. 10.1111/psyp.13038, 29226965

[bib28] Gschwind, M., Michel, C. M., & Van De Ville, D. (2015). Long-range dependencies make the difference—Comment on “A stochastic model for EEG microstate sequence analysis.” NeuroImage, 117, 449–455. 10.1016/j.neuroimage.2015.05.062, 26032884

[bib29] Hipp, J. F., Hawellek, D. J., Corbetta, M., Siegel, M., & Engel, A. K. (2012). Large-scale cortical correlation structure of spontaneous oscillatory activity. Nature Neuroscience, 15(6), 884–890. 10.1038/nn.3101, 22561454 PMC3861400

[bib30] Hipp, J. F., & Siegel, M. (2015). BOLD fMRI correlation reflects frequency-specific neuronal correlation. Current Biology, 25(10), 1368–1374. 10.1016/j.cub.2015.03.049, 25936551

[bib31] Hunyadi, B., Woolrich, M. W., Quinn, A. J., Vidaurre, D., & De Vos, M. (2019). A dynamic system of brain networks revealed by fast transient EEG fluctuations and their fMRI correlates. NeuroImage, 185, 72–82. 10.1016/j.neuroimage.2018.09.082, 30287299

[bib32] Iacono, W. G., Carlson, S. R., Taylor, J., Elkins, I. J., & McGue, M. (1999). Behavioral disinhibition and the development of substance-use disorders: Findings from the Minnesota Twin Family Study. Development and Psychopathology, 11(4), 869–900. 10.1017/S0954579499002369, 10624730

[bib33] Jun, S., Alderson, T. H., Altmann, A., & Sadaghiani, S. (2022). Dynamic trajectories of connectome state transitions are heritable. NeuroImage, 256, 119274. 10.1016/j.neuroimage.2022.119274, 35504564 PMC9223440

[bib34] Jun, S., Malone, S. M., Alderson, T. H., Harper, J., Hunt, R. H., Thomas, K. M., … Sadaghiani, S. (2024). Cognitive abilities are associated with rapid dynamics of electrophysiological connectome states. Network Neuroscience, 8(4), 1089–1104. 10.1162/netn_a_00390, 38293067

[bib35] Kabbara, A., Paban, V., & Hassan, M. (2021). The dynamic modular fingerprints of the human brain at rest. NeuroImage, 227, 117674. 10.1016/j.neuroimage.2020.117674, 33359336

[bib36] Karapanagiotidis, T., Vidaurre, D., Quinn, A. J., Vatansever, D., Poerio, G. L., Turnbull, A., … Smallwood, J. (2020). The psychological correlates of distinct neural states occurring during wakeful rest. Scientific Reports, 10(1), 21121. 10.1038/s41598-020-77336-z, 33273566 PMC7712889

[bib37] Kautzky, A., Baldinger, P., Souery, D., Montgomery, S., Mendlewicz, J., Zohar, J., … Kasper, S. (2015). The combined effect of genetic polymorphisms and clinical parameters on treatment outcome in treatment-resistant depression. European Neuropsychopharmacology, 25(4), 441–453. 10.1016/j.euroneuro.2015.01.001, 25769916

[bib38] Keller, M. C., & Coventry, W. L. (2005). Quantifying and addressing parameter indeterminacy in the classical twin design. Twin Research and Human Genetics, 8(3), 201–213. 10.1375/twin.8.3.201, 15989748

[bib39] Keyes, M. A., Malone, S. M., Elkins, I. J., Legrand, L. N., McGue, M., & Iacono, W. G. (2009). The enrichment study of the Minnesota twin family study: Increasing the yield of twin families at high risk for externalizing psychopathology. Twin Research and Human Genetics, 12(5), 489–501. 10.1375/twin.12.5.489, 19803776 PMC2760025

[bib40] Klaassens, B. L., Rombouts, S. A. R. B., Winkler, A. M., van Gorsel, H. C., van der Grond, J., & van Gerven, J. M. A. (2017). Time related effects on functional brain connectivity after serotonergic and cholinergic neuromodulation. Human Brain Mapping, 38(1), 308–325. 10.1002/hbm.23362, 27622387 PMC5215384

[bib41] Klimesch, W. (2012). Alpha-band oscillations, attention, and controlled access to stored information. Trends in Cognitive Sciences, 16(12), 606–617. 10.1016/j.tics.2012.10.007, 23141428 PMC3507158

[bib42] Kochunov, P., Jahanshad, N., Marcus, D., Winkler, A., Sprooten, E., Nichols, T. E., … Van Essen, D. C. (2015). Heritability of fractional anisotropy in human white matter: A comparison of Human Connectome Project and ENIGMA-DTI data. NeuroImage, 111, 300–311. 10.1016/j.neuroimage.2015.02.050, 25747917 PMC4387079

[bib43] Kong, W., Wang, L., Xu, S., Babiloni, F., & Chen, H. (2019). EEG fingerprints: Phase synchronization of EEG signals as biomarker for subject identification. IEEE Access, 7, 121165–121173. 10.1109/ACCESS.2019.2931624

[bib44] Lehmann, D., Pascual-Marqui, R. D., & Michel, C. (2009). EEG microstates. Scholarpedia, 4(3), 7632. 10.4249/scholarpedia.7632

[bib45] Lord, L.-D., Expert, P., Atasoy, S., Roseman, L., Rapuano, K., Lambiotte, R., … Cabral, J. (2019). Dynamical exploration of the repertoire of brain networks at rest is modulated by psilocybin. NeuroImage, 199, 127–142. 10.1016/j.neuroimage.2019.05.060, 31132450

[bib46] Mognon, A., Jovicich, J., Bruzzone, L., & Buiatti, M. (2011). ADJUST: An automatic EEG artifact detector based on the joint use of spatial and temporal features. Psychophysiology, 48(2), 229–240. 10.1111/j.1469-8986.2010.01061.x, 20636297

[bib47] Newman, M. E. J. (2006). Finding community structure in networks using the eigenvectors of matrices. Physical Review E—Statistical, Nonlinear, and Soft Matter Physics, 74(3), 036104. 10.1103/PhysRevE.74.036104, 17025705

[bib48] Palva, S., & Palva, J. M. (2011). Functional roles of alpha-band phase synchronization in local and large-scale cortical networks. Frontiers in Psychology, 2, 204. 10.3389/fpsyg.2011.00204, 21922012 PMC3166799

[bib49] Posthuma, D., de Geus, E. J. C., Mulder, E. J. C. M., Smit, D. J. A., Boomsma, D. I., & Stam, C. J. (2005). Genetic components of functional connectivity in the brain: The heritability of synchronization likelihood. Human Brain Mapping, 26(3), 191–198. 10.1002/hbm.20156, 15929086 PMC6871713

[bib50] Posthuma, D., Neale, M. C., Boomsma, D. I., & de Geus, E. J. (2001). Are smarter brains running faster? Heritability of alpha peak frequency, IQ, and their interrelation. Behavior Genetics, 31(6), 567–579. 10.1023/A:1013345411774, 11838534

[bib51] Preti, M. G., Bolton, T. A., & Van De Ville, D. (2017). The dynamic functional connectome: State-of-the-art and perspectives. NeuroImage, 160, 41–54. 10.1016/j.neuroimage.2016.12.061, 28034766

[bib52] Quinn, A. J., Vidaurre, D., Abeysuriya, R., Becker, R., Nobre, A. C., & Woolrich, M. W. (2018). Task-evoked dynamic network analysis through hidden Markov modeling. Frontiers in Neuroscience, 12, 603. 10.3389/fnins.2018.00603, 30210284 PMC6121015

[bib53] Rieger, K., Hernandez, L. D., Baenninger, A., & Koenig, T. (2016). 15 years of microstate research in schizophrenia—Where are we? A meta-analysis. Frontiers in Psychiatry, 7, 22. 10.3389/fpsyt.2016.00022, 26955358 PMC4767900

[bib54] Rijsdijk, F. V., & Sham, P. C. (2002). Analytic approaches to twin data using structural equation models. Briefings in Bioinformatics, 3(2), 119–133. 10.1093/bib/3.2.119, 12139432

[bib55] Rousseeuw, P. J., & Croux, C. (1993). Alternatives to the median absolute deviation. Journal of the American Statistical Association, 88(424), 1273–1283. 10.1080/01621459.1993.10476408

[bib56] Rubinov, M., & Sporns, O. (2010). Complex network measures of brain connectivity: Uses and interpretations. NeuroImage, 52(3), 1059–1069. 10.1016/j.neuroimage.2009.10.003, 19819337

[bib57] Sadaghiani, S., & Kleinschmidt, A. (2016). Brain networks and α-oscillations: Structural and functional foundations of cognitive control. Trends in Cognitive Sciences, 20(11), 805–817. 10.1016/j.tics.2016.09.004, 27707588

[bib60] Sadaghiani, S., & Wirsich, J. (2020). Intrinsic connectome organization across temporal scales: New insights from cross-modal approaches. Network Neuroscience, 4(1), 1–29. 10.1162/netn_a_00114, 32043042 PMC7006873

[bib58] Sadaghiani, S., Ng, B., Altmann, A., Poline, J.-B., Banaschewski, T., Bokde, A. L. W., … Greicius, M. (2017). Overdominant effect of a *CHRNA4* polymorphism on cingulo-opercular network activity and cognitive control. Journal of Neuroscience, 37(40), 9657–9666. 10.1523/JNEUROSCI.0991-17.2017, 28877969 PMC6596609

[bib59] Sadaghiani, S., Poline, J.-B., Kleinschmidt, A., & D’Esposito, M. (2015). Ongoing dynamics in large-scale functional connectivity predict perception. Proceedings of the National Academy of Sciences, 112(27), 8463–8468. 10.1073/pnas.1420687112, 26106164 PMC4500238

[bib61] Santarnecchi, E., Khanna, A. R., Musaeus, C. S., Benwell, C. S. Y., Davila, P., Farzan, F., … Honeywell SHARP Team authors. (2017). EEG microstate correlates of fluid intelligence and response to cognitive training. Brain Topography, 30(4), 502–520. 10.1007/s10548-017-0565-z, 28493012

[bib62] Sareen, E., Zahar, S., Van De Ville, D., Gupta, A., Griffa, A., & Amico, E. (2021). Exploring MEG brain fingerprints: Evaluation, pitfalls, and interpretations. NeuroImage, 240, 118331. 10.1016/j.neuroimage.2021.118331, 34237444

[bib63] Schutte, N. M., Hansell, N. K., de Geus, E. J. C., Martin, N. G., Wright, M. J., & Smit, D. J. A. (2013). Heritability of resting state EEG functional connectivity patterns. Twin Research and Human Genetics, 16(5), 962–969. 10.1017/thg.2013.55, 23931641

[bib64] Sha, Z., Schijven, D., Fisher, S. E., & Francks, C. (2023). Genetic architecture of the white matter connectome of the human brain. Science Advances, 9(7), eadd2870. 10.1126/sciadv.add2870, 36800424 PMC9937579

[bib65] Shine, J. M., & Poldrack, R. A. (2018). Principles of dynamic network reconfiguration across diverse brain states. NeuroImage, 180, 396–405. 10.1016/j.neuroimage.2017.08.010, 28782684

[bib66] Shine, J. M., van den Brink, R. L., Hernaus, D., Nieuwenhuis, S., & Poldrack, R. A. (2018). Catecholaminergic manipulation alters dynamic network topology across cognitive states. Network Neuroscience, 2(3), 381–396. 10.1162/netn_a_00042, 30294705 PMC6145851

[bib67] Sinclair, B., Hansell, N. K., Blokland, G. A. M., Martin, N. G., Thompson, P. M., Breakspear, M., … McMahon, K. L. (2015). Heritability of the network architecture of intrinsic brain functional connectivity. NeuroImage, 121, 243–252. 10.1016/j.neuroimage.2015.07.048, 26226088 PMC4837693

[bib68] Sitnikova, T., Hughes, J. W., Howard, C. M., Stephens, K. A., Woolrich, M. W., & Salat, D. H. (2020). Spontaneous activity changes in large-scale cortical networks in older adults couple to distinct hemodynamic morphology. bioRxiv. 10.1101/2020.05.05.079749

[bib69] Smit, C. M., Wright, M. J., Hansell, N. K., Geffen, G. M., & Martin, N. G. (2006). Genetic variation of individual alpha frequency (IAF) and alpha power in a large adolescent twin sample. International Journal of Psychophysiology, 61(2), 235–243. 10.1016/j.ijpsycho.2005.10.004, 16338015

[bib70] Smit, D. J. A., Posthuma, D., Boomsma, D. I., & Geus, E. J. C. (2005). Heritability of background EEG across the power spectrum. Psychophysiology, 42(6), 691–697. 10.1111/j.1469-8986.2005.00352.x, 16364064

[bib71] Smit, D. J. A., Stam, C. J., Posthuma, D., Boomsma, D. I., & de Geus, E. J. C. (2008). Heritability of “small-world” networks in the brain: A graph theoretical analysis of resting-state EEG functional connectivity. Human Brain Mapping, 29(12), 1368–1378. 10.1002/hbm.20468, 18064590 PMC6870849

[bib72] Tadel, F., Baillet, S., Mosher, J. C., Pantazis, D., & Leahy, R. M. (2011). Brainstorm: A user-friendly application for MEG/EEG analysis. Computational Intelligence and Neuroscience, 2011, 879716. 10.1155/2011/879716, 21584256 PMC3090754

[bib73] Tadel, F., Bock, E., Niso, G., Mosher, J. C., Cousineau, M., Pantazis, D., … Baillet, S. (2019). MEG/EEG group analysis with brainstorm. Frontiers in Neuroscience, 13, 76. 10.3389/fnins.2019.00076, 30804744 PMC6378958

[bib74] Tenke, C. E., & Kayser, J. (2001). A convenient method for detecting electrolyte bridges in multichannel electroencephalogram and event-related potential recordings. Clinical Neurophysiology, 112(3), 545–550. 10.1016/S1388-2457(00)00553-8, 11222978

[bib75] Thompson, G. J., Magnuson, M. E., Merritt, M. D., Schwarb, H., Pan, W.-J., McKinley, A., … Keilholz, S. D. (2013). Short-time windows of correlation between large-scale functional brain networks predict vigilance intraindividually and interindividually. Human Brain Mapping, 34(12), 3280–3298. 10.1002/hbm.22140, 22736565 PMC6870033

[bib76] Tost, H., Champagne, F. A., & Meyer-Lindenberg, A. (2015). Environmental influence in the brain, human welfare and mental health. Nature Neuroscience, 18(10), 1421–1431. 10.1038/nn.4108, 26404717

[bib78] van Beijsterveldt, C. E., Molenaar, P. C., de Geus, E. J., & Boomsma, D. I. (1996). Heritability of human brain functioning as assessed by electroencephalography. American Journal of Human Genetics, 58(3), 562–573. 8644716 PMC1914558

[bib77] van Beijsterveldt, C. E., Molenaar, P. C., de Geus, E. J., & Boomsma, D. I. (1998). Genetic and environmental influences on EEG coherence. Behavior Genetics, 28(6), 443–453. 10.1023/A:1021637328512, 9926613

[bib79] Vanderhaeghen, P., & Polleux, F. (2023). Developmental mechanisms underlying the evolution of human cortical circuits. Nature Reviews Neuroscience, 24(4), 213–232. 10.1038/s41583-023-00675-z, 36792753 PMC10064077

[bib80] Van De Ville, D., Britz, J., & Michel, C. M. (2010). EEG microstate sequences in healthy humans at rest reveal scale-free dynamics. Proceedings of the National Academy of Sciences, 107(42), 18179–18184. 10.1073/pnas.1007841107, 20921381 PMC2964192

[bib81] Vidaurre, D., Quinn, A. J., Baker, A. P., Dupret, D., Tejero-Cantero, A., & Woolrich, M. W. (2016). Spectrally resolved fast transient brain states in electrophysiological data. NeuroImage, 126, 81–95. 10.1016/j.neuroimage.2015.11.047, 26631815 PMC4739513

[bib82] Vidaurre, D., Smith, S. M., & Woolrich, M. W. (2017). Brain network dynamics are hierarchically organized in time. Proceedings of the National Academy of Sciences of the United States of America, 114(48), 12827–12832. 10.1073/pnas.1705120114, 29087305 PMC5715736

[bib83] Wilson, S., Haroian, K., Iacono, W. G., Krueger, R. F., Lee, J. J., Luciana, M., … Vrieze, S. (2019). Minnesota center for twin and family research. Twin Research and Human Genetics, 22(6), 746–752. 10.1017/thg.2019.107, 31796137 PMC7056536

[bib84] Wirsich, J., Giraud, A.-L., & Sadaghiani, S. (2020). Concurrent EEG- and fMRI-derived functional connectomes exhibit linked dynamics. NeuroImage, 219, 116998. 10.1016/j.neuroimage.2020.116998, 32480035

[bib85] Wirsich, J., Jorge, J., Iannotti, G. R., Shamshiri, E. A., Grouiller, F., Abreu, R., … Vulliémoz, S. (2021). The relationship between EEG and fMRI connectomes is reproducible across simultaneous EEG-fMRI studies from 1.5T to 7T. NeuroImage, 231, 117864. 10.1016/j.neuroimage.2021.117864, 33592241

[bib86] Wirsich, J., Ridley, B., Besson, P., Jirsa, V., Bénar, C., Ranjeva, J.-P., & Guye, M. (2017). Complementary contributions of concurrent EEG and fMRI connectivity for predicting structural connectivity. NeuroImage, 161, 251–260. 10.1016/j.neuroimage.2017.08.055, 28842386

[bib87] Yashin, A. I., & Iachine, I. A. (1995). Genetic analysis of durations: Correlated frailty model applied to survival of Danish twins. Genetic Epidemiology, 12(5), 529–538. 10.1002/gepi.1370120510, 8557185

[bib88] Yeo, B. T. T., Krienen, F. M., Sepulcre, J., Sabuncu, M. R., Lashkari, D., Hollinshead, M., … Buckner, R. L. (2011). The organization of the human cerebral cortex estimated by intrinsic functional connectivity. Journal of Neurophysiology, 106(3), 1125–1165. 10.1152/jn.00338.2011, 21653723 PMC3174820

[bib89] Zalesky, A., Fornito, A., & Bullmore, E. T. (2010). Network-based statistic: Identifying differences in brain networks. NeuroImage, 53(4), 1197–1207. 10.1016/j.neuroimage.2010.06.041, 20600983

