# Supplementary material for: Rapid dynamics of electrophysiological connectome states are heritable
Source: Netw Neurosci. 2024 Dec 10;8(4):1065–88. doi: 10.1162/netn_a_00391 (PMC11674403; doi:10.1162/netn_a_00391)
Supplement: Supplementary file 1 [file netn-8-4-1065-s001.pdf]

Supplementary Information

Supplementary Tables

Table S1. ANCOVAs of the factor connectome state (K levels) on $FC_{Time\text{-}Varying}$ of all Intrinsic connectivity network (ICN) pairs.

Variables	*K* = 4			*K* = 6		
	***F* states**	***P*†**	**η^2_p**	***F* states**	***P*†**	**η^2_p**
**Delta band**						
$VIS\text{-}SMN_{Time\text{-}Varying}$	8.19E+02	0	0.399	1.98E+02	2.55E-192	0.151
$VIS\text{-}DAN_{Time\text{-}Varying}$	8.62E+01	6.84E-52	0.065	2.60E+02	3.14E-248	0.190
$VIS\text{-}VAN_{Time\text{-}Varying}$	1.12E+03	0	0.475	4.15E+02	0	0.272
$VIS\text{-}Limbic_{Time\text{-}Varying}$	8.96E+02	0	0.421	1.59E+02	9.08E-157	0.126
$VIS\text{-}FPN_{Time\text{-}Varying}$	2.93E+02	9.40E-169	0.192	5.42E+01	4.50E-53	0.047
$VIS\text{-}DMN_{Time\text{-}Varying}$	5.14E+02	2.85E-277	0.295	1.73E+02	2.50E-169	0.135
$SMN\text{-}DAN_{Time\text{-}Varying}$	6.37E+02	0	0.341	5.52E+01	3.72E-54	0.047
$SMN\text{-}VAN_{Time\text{-}Varying}$	3.01E+02	6.06E-173	0.197	6.46E+01	1.04E-63	0.055
$SMN\text{-}Limbic_{Time\text{-}Varying}$	6.41E+02	0	0.342	6.40E+01	4.30E-63	0.055
$SMN\text{-}FPN_{Time\text{-}Varying}$	7.39E+02	0	0.375	8.93E+01	1.40E-88	0.074
$SMN\text{-}DMN_{Time\text{-}Varying}$	1.14E+03	0	0.480	2.13E+02	1.89E-206	0.161
$DAN\text{-}VAN_{Time\text{-}Varying}$	1.07E+02	2.51E-64	0.080	4.59E+02	0	0.293
$DAN\text{-}Limbic_{Time\text{-}Varying}$	1.88E+02	1.82E-111	0.132	4.16E+01	3.74E-40	0.036
$DAN\text{-}FPN_{Time\text{-}Varying}$	4.51E+02	1.28E-247	0.268	1.09E+02	1.20E-107	0.089
$DAN\text{-}DMN_{Time\text{-}Varying}$	8.18E+02	0	0.399	2.18E+02	2.35E-211	0.165
$VAN\text{-}Limbic_{Time\text{-}Varying}$	5.63E+02	1.80E-299	0.314	8.46E+01	6.15E-84	0.071
$VAN\text{-}FPN_{Time\text{-}Varying}$	8.52E+01	2.61E-51	0.065	6.68E+01	6.15E-66	0.057
$VAN\text{-}DMN_{Time\text{-}Varying}$	9.32E+01	3.83E-56	0.070	2.04E+02	6.00E-198	0.155
$Limbic\text{-}FPN_{Time\text{-}Varying}$	8.45E+02	0	0.407	1.04E+02	2.03E-103	0.086
$Limbic\text{-}DMN_{Time\text{-}Varying}$	9.45E+02	0	0.434	1.12E+02	4.97E-111	0.092
$FPN\text{-}DMN_{Time\text{-}Varying}$	1.57E+03	0	0.561	4.94E+02	0	0.308
**Theta band**						
$VIS\text{-}SMN_{Time\text{-}Varying}$	8.37E+02	0	0.405	8.34E+01	1.12E-82	0.070
$VIS\text{-}DAN_{Time\text{-}Varying}$	2.36E+00	7.31E+00	0.002	3.23E+01	1.47E-30	0.028
$VIS\text{-}VAN_{Time\text{-}Varying}$	1.24E+03	0	0.502	3.57E+02	0	0.244
$VIS\text{-}Limbic_{Time\text{-}Varying}$	9.70E+02	0	0.441	1.53E+02	5.77E-151	0.122
$VIS\text{-}FPN_{Time\text{-}Varying}$	3.29E+02	3.82E-187	0.211	3.35E+01	9.63E-32	0.029
$VIS\text{-}DMN_{Time\text{-}Varying}$	5.52E+02	3.74E-294	0.310	7.12E+01	2.29E-70	0.060
$SMN\text{-}DAN_{Time\text{-}Varying}$	3.58E+02	4.19E-202	0.226	1.49E+02	5.67E-147	0.119
$SMN\text{-}VAN_{Time\text{-}Varying}$	3.58E+02	4.15E-202	0.226	5.62E+01	3.88E-55	0.048
$SMN\text{-}Limbic_{Time\text{-}Varying}$	7.16E+02	0	0.368	5.58E+01	8.53E-55	0.048
$SMN\text{-}FPN_{Time\text{-}Varying}$	9.89E+02	0	0.446	1.69E+02	9.89E-166	0.132
$SMN\text{-}DMN_{Time\text{-}Varying}$	1.05E+03	0	0.461	2.09E+02	2.14E-202	0.159
$DAN\text{-}VAN_{Time\text{-}Varying}$	3.91E+01	7.70E-23	0.031	8.20E+01	2.99E-81	0.069
$DAN\text{-}Limbic_{Time\text{-}Varying}$	7.65E+01	5.72E-46	0.059	1.97E+01	1.54E-17	0.018
$DAN\text{-}FPN_{Time\text{-}Varying}$	2.24E+02	1.14E-131	0.154	6.18E+01	7.18E-61	0.053

DAN-DMN$_{\text{Time-Varying}}$	3.90E+02	7.51E-218	0.241	1.08E+02	1.19E-106	0.089
VAN-Limbic$_{\text{Time-Varying}}$	5.68E+02	2.11E-301	0.316	1.20E+02	1.28E-118	0.098
VAN-FPN$_{\text{Time-Varying}}$	8.86E+01	2.54E-53	0.067	3.31E+01	2.01E-31	0.029
VAN-DMN$_{\text{Time-Varying}}$	1.07E+02	1.06E-64	0.080	4.50E+01	1.27E-43	0.039
Limbic-FPN$_{\text{Time-Varying}}$	6.17E+02	0	0.334	5.21E+01	6.32E-51	0.045
Limbic-DMN$_{\text{Time-Varying}}$	8.96E+02	0	0.421	1.12E+02	2.25E-111	0.092
FPN-DMN$_{\text{Time-Varying}}$	9.14E+02	0	0.426	2.71E+02	7.07E-258	0.197
**Alpha band**						
VIS-SMN$_{\text{Time-Varying}}$	9.45E+01	6.69E-57	0.071	6.42E+02	0	0.367
VIS-DAN$_{\text{Time-Varying}}$	2.24E+02	2.79E-131	0.154	1.05E+02	2.71E-104	0.087
VIS-VAN$_{\text{Time-Varying}}$	6.35E+02	0	0.340	4.99E+02	0	0.311
VIS-Limbic$_{\text{Time-Varying}}$	8.22E+01	1.81E-49	0.063	4.61E+02	0	0.294
VIS-FPN$_{\text{Time-Varying}}$	1.48E+01	1.44E-07	0.012	3.49E+02	0	0.240
VIS-DMN$_{\text{Time-Varying}}$	5.64E+01	1.33E-33	0.044	6.75E+02	0	0.379
SMN-DAN$_{\text{Time-Varying}}$	3.78E+02	3.72E-212	0.235	2.29E+02	9.55E-221	0.171
SMN-VAN$_{\text{Time-Varying}}$	5.86E+01	5.40E-35	0.045	4.26E+02	0	0.278
SMN-Limbic$_{\text{Time-Varying}}$	1.72E+02	1.82E-102	0.123	6.30E+02	0	0.363
SMN-FPN$_{\text{Time-Varying}}$	1.98E+02	4.49E-117	0.139	5.37E+02	0	0.327
SMN-DMN$_{\text{Time-Varying}}$	3.03E+02	1.17E-173	0.197	8.00E+02	0	0.419
DAN-VAN$_{\text{Time-Varying}}$	4.61E+02	1.80E-252	0.273	9.53E+01	1.34E-94	0.079
DAN-Limbic$_{\text{Time-Varying}}$	1.84E+02	6.45E-109	0.130	1.04E+02	4.92E-103	0.086
DAN-FPN$_{\text{Time-Varying}}$	2.94E+02	2.32E-169	0.193	1.60E+02	7.36E-157	0.126
DAN-DMN$_{\text{Time-Varying}}$	4.44E+02	4.06E-244	0.265	2.98E+02	2.80E-281	0.212
VAN-Limbic$_{\text{Time-Varying}}$	4.10E+01	5.21E-24	0.032	4.15E+02	0	0.272
VAN-FPN$_{\text{Time-Varying}}$	8.46E+01	6.40E-51	0.064	2.31E+02	2.82E-222	0.172
VAN-DMN$_{\text{Time-Varying}}$	6.97E+01	8.06E-42	0.054	4.06E+02	0	0.268
Limbic-FPN$_{\text{Time-Varying}}$	1.95E+02	2.16E-115	0.137	3.94E+02	0	0.262
Limbic-DMN$_{\text{Time-Varying}}$	2.02E+02	3.02E-119	0.141	7.10E+02	0	0.391
FPN-DMN$_{\text{Time-Varying}}$	4.28E+02	1.83E-236	0.258	6.60E+02	0	0.373
**Beta band**						
VIS-SMN$_{\text{Time-Varying}}$	4.51E+02	1.39E-247	0.268	2.43E+02	2.19E-228	0.211
VIS-DAN$_{\text{Time-Varying}}$	1.20E+02	3.13E-72	0.089	8.33E+01	6.01E-82	0.084
VIS-VAN$_{\text{Time-Varying}}$	7.26E+02	0	0.371	2.71E+02	4.67E-252	0.230
VIS-Limbic$_{\text{Time-Varying}}$	3.34E+02	5.78E-190	0.214	2.50E+02	7.00E-235	0.216
VIS-FPN$_{\text{Time-Varying}}$	4.44E+01	3.56E-26	0.035	8.69E+01	1.70E-85	0.087
VIS-DMN$_{\text{Time-Varying}}$	1.52E+02	5.16E-91	0.110	2.36E+02	9.32E-223	0.206
SMN-DAN$_{\text{Time-Varying}}$	3.51E+02	1.62E-198	0.222	n/a	n/a	n/a
SMN-VAN$_{\text{Time-Varying}}$	1.49E+02	3.95E-89	0.108	1.14E+02	7.33E-112	0.112
SMN-Limbic$_{\text{Time-Varying}}$	2.79E+02	3.68E-161	0.185	n/a	n/a	n/a
SMN-FPN$_{\text{Time-Varying}}$	4.80E+02	2.43E-261	0.281	n/a	n/a	n/a
SMN-DMN$_{\text{Time-Varying}}$	6.24E+02	0	0.337	5.06E+02	0	0.358
DAN-VAN$_{\text{Time-Varying}}$	1.84E+02	5.36E-109	0.130	n/a	n/a	n/a
DAN-Limbic$_{\text{Time-Varying}}$	7.53E+01	2.87E-45	0.058	n/a	n/a	n/a
DAN-FPN$_{\text{Time-Varying}}$	2.93E+02	1.66E-168	0.192	n/a	n/a	n/a
DAN-DMN$_{\text{Time-Varying}}$	4.70E+02	8.22E-257	0.277	3.12E+02	7.98E-286	0.256
VAN-Limbic$_{\text{Time-Varying}}$	2.27E+02	4.74E-133	0.156	1.98E+02	3.52E-189	0.179

VAN-FPN$_{Time-Varying}$	1.64E+01	1.43E-08	0.013	3.76E+01	7.03E-36	0.040
VAN-DMN$_{Time-Varying}$	4.70E+01	8.94E-28	0.037	9.89E+01	3.32E-97	0.098
Limbic-FPN$_{Time-Varying}$	3.87E+02	2.12E-216	0.239	1.68E+02	6.23E-162	0.156
Limbic-DMN$_{Time-Varying}$	3.77E+02	8.99E-212	0.235	3.02E+02	5.66E-278	0.250
FPN-DMN$_{Time-Varying}$	9.01E+02	0	0.423	3.77E+02	0	0.293
**Gamma band**						
VIS-SMN$_{Time-Varying}$	4.85E+02	9.78E-264	0.283	2.87E+02	1.57E-270	0.212
VIS-DAN$_{Time-Varying}$	4.55E+01	7.36E-27	0.036	3.25E+01	8.82E-31	0.030
VIS-VAN$_{Time-Varying}$	6.04E+02	3.19E-317	0.330	3.41E+02	9.12e-316	0.242
VIS-Limbic$_{Time-Varying}$	4.62E+02	6.87E-253	0.273	2.77E+02	3.06E-262	0.206
VIS-FPN$_{Time-Varying}$	1.86E+02	2.90E-110	0.131	8.55E+01	1.09E-84	0.074
VIS-DMN$_{Time-Varying}$	2.45E+02	6.52E-143	0.166	1.15E+02	1.33E-113	0.097
SMN-DAN$_{Time-Varying}$	6.51E+02	0	0.347	3.20E+02	2.07E-298	0.231
SMN-VAN$_{Time-Varying}$	2.08E+02	8.50E-123	0.145	1.17E+02	4.60E-116	0.099
SMN-Limbic$_{Time-Varying}$	6.05E+02	1.35e-317	0.330	3.23E+02	1.08E-300	0.232
SMN-FPN$_{Time-Varying}$	5.99E+02	5.68e-315	0.328	n/a	n/a	n/a
SMN-DMN$_{Time-Varying}$	7.37E+02	0	0.375	5.13E+02	0	0.324
DAN-VAN$_{Time-Varying}$	7.22E+01	2.48E-43	0.055	2.03E+01	4.26E-18	0.019
DAN-Limbic$_{Time-Varying}$	2.05E+02	4.44E-121	0.143	1.04E+02	9.55E-103	0.089
DAN-FPN$_{Time-Varying}$	4.79E+02	1.27E-260	0.280	n/a	n/a	n/a
DAN-DMN$_{Time-Varying}$	8.87E+02	0	0.419	4.65E+02	0	0.303
VAN-Limbic$_{Time-Varying}$	3.71E+02	1.05E-208	0.232	2.24E+02	9.24E-216	0.174
VAN-FPN$_{Time-Varying}$	8.48E+01	5.12E-51	0.065	2.28E+01	1.06E-20	0.021
VAN-DMN$_{Time-Varying}$	7.52E+01	3.50E-45	0.058	2.16E+01	1.80E-19	0.020
Limbic-FPN$_{Time-Varying}$	6.61E+02	0	0.350	3.07E+02	2.69E-287	0.223
Limbic-DMN$_{Time-Varying}$	5.88E+02	2.13e-310	0.324	3.01E+02	2.64E-282	0.220
FPN-DMN$_{Time-Varying}$	9.98E+02	0	0.448	6.09E+02	0	0.363

We performed one-way ANCOVAs of the factor connectome state on each FCTime-Varying between ICN pairs, adjusted for age and sex. K: the chosen number of discrete connectome states, P†: P values Bonferroni-corrected for 105 tests (21 dependent variables of all ICN pairs and five frequency bands), η2p: Partial Eta squared effect size, n/a: not applicable.

Table S2. ANCOVAs of the factor connectome state (*K* levels) on $\text{Modularity}_{\text{Time-Varying}}$ and Fractional Occupancy

**Variables**	***K* = 4**			***K* = 6**		
	***F* states**	$P^{\dagger}$	η^2_p	***F* states**	$P^{\dagger}$	η^2_p
Delta band						
$\text{Modularity}_{\text{Time-Varying}}$	1.41E+02	2.68E-85	0.103	3.47E+02	0	0.238
Fractional Occupancy	5.16E+03	0	0.807	2.43E+03	0	0.686
Theta band						
$\text{Modularity}_{\text{Time-Varying}}$	8.67E+01	6.33E-53	0.066	1.98E+02	3.16E-193	0.152
Fractional Occupancy	9.30E+02	0	0.430	1.16E+03	0	0.510
Alpha band						
$\text{Modularity}_{\text{Time-Varying}}$	6.22E+01	6.38E-38	0.048	8.00E+01	5.11E-80	0.067
Fractional Occupancy	1.86E+03	0	0.601	1.14E+03	0	0.507
Beta band						
$\text{Modularity}_{\text{Time-Varying}}$	3.30E+02	1.60E-188	0.212	1.08E+02	9.47E-107	0.105
Fractional Occupancy	7.20E+02	0	0.369	2.05E+03	0	0.649
Gamma band						
$\text{Modularity}_{\text{Time-Varying}}$	1.55E+02	2.29E-93	0.112	6.48E+01	1.33E-64	0.057
Fractional Occupancy	5.44E+02	1.06E-29	0.306	8.89E+02	0	0.445

We performed one-way ANCOVAs of the factor connectome state on $\text{Modularity}_{\text{Time-Varying}}$ and Fractional Occupancy, adjusted for age and sex. *K*: the chosen number of discrete connectome states, $P^{\dagger}$: *P* values Bonferroni-corrected for 20 tests (four main dependent variables and five frequency bands), η^2_p: Partial Eta squared effect size.

Table S3. ANCOVAs of the factor sibling status (*3* levels) on $\text{Modularity}_{\text{Time-Varying}}$ and $\text{FC}_{\text{Time-Varying}}$ of data-driven clusters with different initial connection-wise thresholds (density).

	K = 6				***K = 4***			
**Phenotypes**	***F* sibling status**	***P*†**	η^2_p	**BF_{01}**	***F* sibling status**	***P*†**	η^2_p	**BF_{01}**
**Delta (1-3 Hz)**								
$\text{Modularity}_{\text{Time-Varying}}$	.08	1.00	<.001	36.79	5.27	.108	.023	.231
NBS-$\text{FC}_{\text{Time-Varying}}$ 5%	1.122	1.00	0.005	13.81	0.869	1.00	0.004	17.65
NBS-$\text{FC}_{\text{Time-Varying}}$ 4%	n/a	n/a	n/a	n/a	0.792	1.00	0.003	18.69
NBS-$\text{FC}_{\text{Time-Varying}}$ 3%	1.981	1.00	0.009	5.72	0.72	1.00	0.003	20.08
NBS-$\text{FC}_{\text{Time-Varying}}$ 2%	1.989	1.00	0.009	6.34	1.586	1.00	0.007	8.93
NBS-$\text{FC}_{\text{Time-Varying}}$ 1%	1.152	1.00	0.005	13.79	0.004	1.00	1.67e-05	39.54
**Theta (4-7 Hz)**								
$\text{Modularity}_{\text{Time-Varying}}$	2.88	1.00	.012	2.90	.768	1.00	.003	17.79
NBS-$\text{FC}_{\text{Time-Varying}}$ 5%	1.252	1.00	0.005	12.41	0.647	1.00	0.003	21.74
NBS-$\text{FC}_{\text{Time-Varying}}$ 4%	1.529	1.00	0.007	9.61	0.393	1.00	0.002	27.42
NBS-$\text{FC}_{\text{Time-Varying}}$ 3%	1.066	1.00	0.005	14.62	0.289	1.00	0.001	29.69
NBS-$\text{FC}_{\text{Time-Varying}}$ 2%	0.719	1.00	0.003	19.90	0.86	1.00	0.004	17.45
NBS-$\text{FC}_{\text{Time-Varying}}$ 1%	1.379	1.00	0.006	10.55	0.902	1.00	0.004	16.60
**Alpha (8-12 Hz)**								
$\text{Modularity}_{\text{Time-Varying}}$	3.57	.577	.015	1.72	.300	1.00	.001	31.65
NBS-$\text{FC}_{\text{Time-Varying}}$ 5%	0.181	1.00	7.91e-04	33.57	0.206	1.00	8.98e-04	32.61
NBS-$\text{FC}_{\text{Time-Varying}}$ 4%	0.119	1.00	5.18e-04	35.66	0.129	1.00	5.61e-04	34.99
NBS-$\text{FC}_{\text{Time-Varying}}$ 3%	0.11	1.00	4.82e-04	36.58	0.164	1.00	7.14e-04	33.30
NBS-$\text{FC}_{\text{Time-Varying}}$ 2%	n/a	n/a	n/a	n/a	0.02	1.00	8.70e-05	38.49
NBS-$\text{FC}_{\text{Time-Varying}}$ 1%	0.24	1.00	0.001	32.93	0.96	1.00	0.004	14.22
**Beta (13-25 Hz)**								
$\text{Modularity}_{\text{Time-Varying}}$	.93	1.00	.004	17.05	1.05	1.00	.005	14.97
NBS-$\text{FC}_{\text{Time-Varying}}$ 5%	0.076	1.00	3.33e-04	37.01	1.314	1.00	0.006	11.52
NBS-$\text{FC}_{\text{Time-Varying}}$ 4%	0.092	1.00	4.02e-04	36.48	1.749	1.00	0.008	7.65
NBS-$\text{FC}_{\text{Time-Varying}}$ 3%	0.086	1.00	3.76e-04	36.85	n/a	n/a	n/a	n/a
NBS-$\text{FC}_{\text{Time-Varying}}$ 2%	0.144	1.00	6.28e-04	35.16	2.581	1.00	0.011	3.74
NBS-$\text{FC}_{\text{Time-Varying}}$ 1%	0.128	1.00	5.60e-04	36.05	0.558	1.00	0.002	23.41
**Gamma (30-45 Hz)**								
$\text{Modularity}_{\text{Time-Varying}}$	.45	1.00	.002	23.88	.126	1.00	.001	35.25
NBS-$\text{FC}_{\text{Time-Varying}}$ 5%	0.182	1.00	7.95e-04	33.64	n/a	n/a	n/a	n/a
NBS-$\text{FC}_{\text{Time-Varying}}$ 4%	n/a	n/a	n/a	n/a	1.1	1.00	0.005	13.33
NBS-$\text{FC}_{\text{Time-Varying}}$ 3%	0.201	1.00	8.76e-04	33.72	1.104	1.00	0.005	13.19
NBS-$\text{FC}_{\text{Time-Varying}}$ 2%	0.158	1.00	6.88e-04	35.32	1.101	1.00	0.005	13.10
NBS-$\text{FC}_{\text{Time-Varying}}$ 1%	0.502	1.00	0.002	24.93	2.067	1.00	0.009	5.38

We performed Network-Based Statistics (NBS) to select connected sets of connections (i.e., clusters) in a data-driven manner (Zalesky et al., 2010). We started with the absolute functional connectivity (FC) matrices, where all connections were transformed into absolute values to focus on the strength of connections, regardless of their connectivity direction. NBS implements an ANCOVA of the factor state for every connection and quantifies the degree to which each

connection's connectivity strength differed across states, adjusted for age and head motion. The ensuing connection-wise *F* statistics was thresholded at arbitrary values, allowing 1 ~ 5% of connections to survive. The multivariate spatial features were constructed as $1 \times K$ vector by averaging the absolute FC values of all connections of the cluster for each state. *F* values are reported for one-way ANCOVAs of the factor sibling status (three levels: monozygotic twins (MZ), sex-matched dizygotic twins (DZ), and sex-matched pairs of unrelated individuals), adjusted for age and sex. *K*: the chosen number of discrete connectome states, $P^{\dagger}$: *P* values Bonferroni-corrected for 25 tests for NBS-$FC_{Time\text{-}Varying}$ measures (five density levels and five frequency bands; capped at 1 maximum) and for 20 tests for $Modularity_{Time\text{-}Varying}$ (four main multivariate features and five frequency bands), η^2_p: Partial Eta squared effect size, BF_{01}: the probability of H_0 against H_1.

Table S4. ANCOVAs of the factor sibling status (*3* levels) on $FC_{Time\text{-}Varying}$ of all Intrinsic connectivity network (ICN) pairs.

**Variables**	***K* = 4**			***K* = 6**		
	***F* sibling status**	***P*†**	η^2_p	***F* sibling status**	***P*†**	η^2_p
**Delta band**						
$VIS\text{-}SMN_{Time\text{-}Varying}$	0.259	1	0.001	0.358	1	0.002
$VIS\text{-}DAN_{Time\text{-}Varying}$	1.146	1	0.005	0.215	1	9.39e-04
$VIS\text{-}VAN_{Time\text{-}Varying}$	0.031	1	1.36e-04	0.148	1	6.47e-04
$VIS\text{-}Limbic_{Time\text{-}Varying}$	0.132	1	5.77e-04	0.323	1	0.001
$VIS\text{-}FPN_{Time\text{-}Varying}$	0.298	1	0.001	0.088	1	3.84e-04
$VIS\text{-}DMN_{Time\text{-}Varying}$	0.633	1	0.003	0.65	1	0.003
$SMN\text{-}DAN_{Time\text{-}Varying}$	1.213	1	0.005	0.632	1	0.003
$SMN\text{-}VAN_{Time\text{-}Varying}$	0.454	1	0.002	0.125	1	5.47e-04
$SMN\text{-}Limbic_{Time\text{-}Varying}$	2.168	1	0.009	0.428	1	0.002
$SMN\text{-}FPN_{Time\text{-}Varying}$	0.201	1	8.80e-04	1.133	1	0.005
$SMN\text{-}DMN_{Time\text{-}Varying}$	1.524	1	0.007	1.613	1	0.007
$DAN\text{-}VAN_{Time\text{-}Varying}$	0.327	1	0.001	1.325	1	0.006
$DAN\text{-}Limbic_{Time\text{-}Varying}$	0.372	1	0.002	0.262	1	0.001
$DAN\text{-}FPN_{Time\text{-}Varying}$	0.369	1	0.002	0.073	1	3.17e-04
$DAN\text{-}DMN_{Time\text{-}Varying}$	0.257	1	0.001	0.904	1	0.004
$VAN\text{-}Limbic_{Time\text{-}Varying}$	0.993	1	0.004	0.926	1	0.004
$VAN\text{-}FPN_{Time\text{-}Varying}$	0.475	1	0.002	0.86	1	0.004
$VAN\text{-}DMN_{Time\text{-}Varying}$	0.963	1	0.004	3.003	1	0.013
$Limbic\text{-}FPN_{Time\text{-}Varying}$	0.548	1	0.002	0.109	1	4.77e-04
$Limbic\text{-}DMN_{Time\text{-}Varying}$	0.636	1	0.003	0.566	1	0.002
$FPN\text{-}DMN_{Time\text{-}Varying}$	0.194	1	8.52e-04	0.194	1	8.45e-04
**Theta band**						
$VIS\text{-}SMN_{Time\text{-}Varying}$	2.354	1	0.01	3.185	1	0.014
$VIS\text{-}DAN_{Time\text{-}Varying}$	0.029	1	1.29e-04	0.074	1	3.25e-04
$VIS\text{-}VAN_{Time\text{-}Varying}$	0.019	1	8.57e-05	1.089	1	0.005
$VIS\text{-}Limbic_{Time\text{-}Varying}$	1.088	1	0.005	0.674	1	0.003
$VIS\text{-}FPN_{Time\text{-}Varying}$	0.279	1	0.001	0.573	1	0.002
$VIS\text{-}DMN_{Time\text{-}Varying}$	1.416	1	0.006	1.909	1	0.008
$SMN\text{-}DAN_{Time\text{-}Varying}$	0.317	1	0.001	0.361	1	0.002
$SMN\text{-}VAN_{Time\text{-}Varying}$	0.255	1	0.001	0.986	1	0.004
$SMN\text{-}Limbic_{Time\text{-}Varying}$	2.478	1	0.011	0.71	1	0.003
$SMN\text{-}FPN_{Time\text{-}Varying}$	0.147	1	6.52e-04	0.069	1	3.01e-04
$SMN\text{-}DMN_{Time\text{-}Varying}$	0.174	1	7.70e-04	0.175	1	7.62e-04
$DAN\text{-}VAN_{Time\text{-}Varying}$	1.339	1	0.006	0.812	1	0.004
$DAN\text{-}Limbic_{Time\text{-}Varying}$	1.864	1	0.008	1.878	1	0.008
$DAN\text{-}FPN_{Time\text{-}Varying}$	0.225	1	9.94e-04	0.413	1	0.002
$DAN\text{-}DMN_{Time\text{-}Varying}$	0.825	1	0.004	0.241	1	0.001
$VAN\text{-}Limbic_{Time\text{-}Varying}$	0.631	1	0.003	0.096	1	4.20e-04
$VAN\text{-}FPN_{Time\text{-}Varying}$	0.482	1	0.002	0.446	1	0.002
$VAN\text{-}DMN_{Time\text{-}Varying}$	0.2	1	8.84e-04	0.488	1	0.002

Limbic-FPN$_{\text{Time-Varying}}$	2.413	1	0.011	0.051	1	2.23e-04
Limbic-DMN$_{\text{Time-Varying}}$	0.072	1	3.18e-04	1.063	1	0.005
FPN-DMN$_{\text{Time-Varying}}$	0.149	1	6.57e-04	0.706	1	0.003
**Alpha band**						
VIS-SMN$_{\text{Time-Varying}}$	3.577	1	0.016	0.413	1	0.002
VIS-DAN$_{\text{Time-Varying}}$	0.172	1	7.60e-04	0.454	1	0.002
VIS-VAN$_{\text{Time-Varying}}$	6.649	0	0.029	0.172	1	7.51e-04
VIS-Limbic$_{\text{Time-Varying}}$	4.54	1	0.02	0.041	1	1.79e-04
VIS-FPN$_{\text{Time-Varying}}$	3.29	1	0.014	0.453	1	0.002
VIS-DMN$_{\text{Time-Varying}}$	5.158	0.6	0.022	1.278	1	0.006
SMN-DAN$_{\text{Time-Varying}}$	1.451	1	0.006	0.851	1	0.004
SMN-VAN$_{\text{Time-Varying}}$	1.886	1	0.008	1.379	1	0.006
SMN-Limbic$_{\text{Time-Varying}}$	2.299	1	0.01	0.475	1	0.002
SMN-FPN$_{\text{Time-Varying}}$	3.041	1	0.013	1.106	1	0.005
SMN-DMN$_{\text{Time-Varying}}$	4.311	1	0.019	3.599	1	0.015
DAN-VAN$_{\text{Time-Varying}}$	0.463	1	0.002	0.738	1	0.003
DAN-Limbic$_{\text{Time-Varying}}$	0.046	1	2.03e-04	1.068	1	0.005
DAN-FPN$_{\text{Time-Varying}}$	0.907	1	0.004	1.015	1	0.004
DAN-DMN$_{\text{Time-Varying}}$	1.799	1	0.008	0.12	1	5.25e-04
VAN-Limbic$_{\text{Time-Varying}}$	0.514	1	0.002	0.72	1	0.003
VAN-FPN$_{\text{Time-Varying}}$	1.033	1	0.005	0.74	1	0.003
VAN-DMN$_{\text{Time-Varying}}$	5.086	0.6	0.022	1.741	1	0.008
Limbic-FPN$_{\text{Time-Varying}}$	1.854	1	0.008	0.724	1	0.003
Limbic-DMN$_{\text{Time-Varying}}$	4.73	1	0.02	0.018	1	7.94e-05
FPN-DMN$_{\text{Time-Varying}}$	1.214	1	0.005	0.185	1	8.06e-04
**Beta band**						
VIS-SMN$_{\text{Time-Varying}}$	2.016	1	0.009	1.933	1	0.008
VIS-DAN$_{\text{Time-Varying}}$	1.149	1	0.005	0.344	1	0.001
VIS-VAN$_{\text{Time-Varying}}$	1.104	1	0.005	0.209	1	9.11e-04
VIS-Limbic$_{\text{Time-Varying}}$	1.613	1	0.007	0.54	1	0.002
VIS-FPN$_{\text{Time-Varying}}$	1.199	1	0.005	0.605	1	0.003
VIS-DMN$_{\text{Time-Varying}}$	1.792	1	0.008	1.378	1	0.006
SMN-DAN$_{\text{Time-Varying}}$	1.987	1	0.009	n/a	n/a	n/a
SMN-VAN$_{\text{Time-Varying}}$	1.072	1	0.005	0.053	1	2.29e-04
SMN-Limbic$_{\text{Time-Varying}}$	0.319	1	0.001	0.056	1	2.43e-04
SMN-FPN$_{\text{Time-Varying}}$	0.378	1	0.002	n/a	n/a	n/a
SMN-DMN$_{\text{Time-Varying}}$	0.312	1	0.001	0.878	1	0.004
DAN-VAN$_{\text{Time-Varying}}$	1.628	1	0.007	n/a	n/a	n/a
DAN-Limbic$_{\text{Time-Varying}}$	0.04	1	1.78e-04	n/a	n/a	n/a
DAN-FPN$_{\text{Time-Varying}}$	1.474	1	0.006	n/a	n/a	n/a
DAN-DMN$_{\text{Time-Varying}}$	0.036	1	1.60e-04	0.37	1	0.002
VAN-Limbic$_{\text{Time-Varying}}$	1.39	1	0.006	0.119	1	5.17e-04
VAN-FPN$_{\text{Time-Varying}}$	0.453	1	0.002	1.46	1	0.006
VAN-DMN$_{\text{Time-Varying}}$	0.346	1	0.002	1.014	1	0.004
Limbic-FPN$_{\text{Time-Varying}}$	0.19	1	8.42e-04	2.052	1	0.009
Limbic-DMN$_{\text{Time-Varying}}$	0.231	1	0.001	0.183	1	7.99e-04

FPN-DMN$_{Time-Varying}$	0.03	1	1.31e-04	0.522	1	0.002
**Gamma band**						
VIS-SMN$_{Time-Varying}$	2.118	1	0.009	0.383	1	0.002
VIS-DAN$_{Time-Varying}$	1.381	1	0.006	0.125	1	5.46e-04
VIS-VAN$_{Time-Varying}$	0.516	1	0.002	5.537	0.6	0.024
VIS-Limbic$_{Time-Varying}$	0.909	1	0.004	1.148	1	0.005
VIS-FPN$_{Time-Varying}$	3.499	1	0.015	1.027	1	0.004
VIS-DMN$_{Time-Varying}$	1.591	1	0.007	0.956	1	0.004
SMN-DAN$_{Time-Varying}$	0.883	1	0.004	0.091	1	3.96e-04
SMN-VAN$_{Time-Varying}$	0.134	1	5.85e-04	0.303	1	0.001
SMN-Limbic$_{Time-Varying}$	1.044	1	0.005	0.154	1	6.71e-04
SMN-FPN$_{Time-Varying}$	0.862	1	0.004	0.401	1	0.002
SMN-DMN$_{Time-Varying}$	1.189	1	0.005	0.475	1	0.002
DAN-VAN$_{Time-Varying}$	2.565	1	0.011	2.174	1	0.009
DAN-Limbic$_{Time-Varying}$	0.692	1	0.003	0.383	1	0.002
DAN-FPN$_{Time-Varying}$	2.293	1	0.01	n/a	n/a	n/a
DAN-DMN$_{Time-Varying}$	2.021	1	0.009	1.438	1	0.006
VAN-Limbic$_{Time-Varying}$	1.438	1	0.006	0.695	1	0.003
VAN-FPN$_{Time-Varying}$	1.925	1	0.008	1.055	1	0.005
VAN-DMN$_{Time-Varying}$	0.955	1	0.004	2.546	1	0.011
Limbic-FPN$_{Time-Varying}$	0.854	1	0.004	0.078	1	3.39e-04
Limbic-DMN$_{Time-Varying}$	0.234	1	0.001	2.408	1	0.01
FPN-DMN$_{Time-Varying}$	4.815	1	0.021	0.944	1	0.004

Heritability of each of the FC$_{Time-Varying}$ between ICN pairs was assessed separately by one-way ANCOVAs of the factor sibling status (three levels: monozygotic twins (MZ), sex-matched dizygotic twins (DZ), and sex-matched pairs of unrelated individuals), adjusted for age and sex. The main effect of sibling status indicates the heritability, or genetic effect. *K*: the chosen number of discrete connectome states, $P^{\dagger}$: *P* values Bonferroni-corrected for 105 tests (5 frequency bands and 21 dependent variables of all ICN pairs), η^2_p: Partial Eta squared effect size.

Table S5. Heritability of individual components of temporal dynamic connectome features.

Frequency band	Variables	*F* sibling status	$P^{\dagger}$ value	η^2_p	*F interaction*	$P^{\dagger}$ value
***K* = 4**						
Delta band	Transition Probability	11.84	7.39e-05	0.004	1.83	.104
	Fractional Occupancy	12.06	6.29e-05	0.013	.207	1.00
Theta band	Transition Probability	21.79	3.76e-09	0.008	2.66	4.07e-04
	Fractional Occupancy	19.73	3.34e-08	0.021	.691	1.00
Alpha band	Transition Probability	121.08	3.50e-51	0.042	2.87	8.44e-05
	Fractional Occupancy	23.29	1.03e-09	0.025	.951	1.00
Beta band	Transition Probability	38.37	2.83e-16	0.014	4.09	4.24e-09
	Fractional Occupancy	24.18	4.33e-10	0.026	2.02	.603
Gamma band	Transition Probability	27.68	1.09e-11	0.01	4.09	4.31e-09
	Fractional Occupancy	23.45	8.81e-10	0.025	2.77	.111
***K* = 6**						
Delta band	Transition Probability	23.17	9.03e-10	0.003	1.39	.264
	Fractional Occupancy	17.32	3.34e-07	0.012	.621	1
Theta band	Transition Probability	89.08	3.67e-38	0.013	1.37	.314
	Fractional Occupancy	33.34	4.92e-14	0.024	1.23	1
Alpha band	Transition Probability	127.01	2.20e-54	0.018	3.61	8.53e-18
	Fractional Occupancy	51.79	8.33e-22	0.036	2.29	.114
Beta band	Transition Probability	32.61	7.44e-14	0.005	3.81	1.28e-19
	Fractional Occupancy	25.37	1.21e-10	0.018	3.60	9.16e-04
Gamma band	Transition Probability	69.27	1.16e-29	0.01	2.52	1.77e-08
	Fractional Occupancy	25.19	1.44e-10	0.018	3.44	1.69e-03

A two-way ANCOVA was conducted to examine the effect of sibling status on the *state-wise* similarity between subject pairs for each temporal feature. Specifically, the factors comprised sibling status (four levels: monozygotic twins (MZ), sex-matched dizygotic twins (DZ), and sex-matched pairs of unrelated individuals) and connectome states or state pairs (*K*=4 or 6 states for Fractional Occupancy and 12 or 30 state pairs for Transition Probability, respectively), adjusted for age and sex. *F* values are reported for the factor sibling status. The effect sizes (h^2_p) for the factor sibling status were smaller than those of the multivariate temporal features in the main analyses (cf. Figure 3). Interestingly, Transition Probability showed significant interaction effects, irrespective of the number of Ks and most of the frequency bands. This implies that heritability is dependent on specific pair of Transition Probability between connectome states. For completeness, we also note that, non-surprisingly, there were significant main effects of the factor connectome states, implying that the similarity across pairs of subjects varied by state. K: the chosen number of discrete connectome states, $P^{\dagger}$: *P* values Bonferroni-corrected for 10 tests (2 dependent variables and five frequency bands), η^2_p: Partial Eta squared effect size of the sibling status.

Table S6. Variance-component model parameter estimates of the dynamic connectome features (K = 4).

**Phenotypes**	**Genetic model**	***h*²**	**(95% CI)**	**A**	**(95% CI)**	**C/D**	**(95% CI)**	**E**	**(95% CI)**	**-2LL**	**AIC**	**chi**	**df**	**p(chi)**
**Delta band**														
Fractional Occupancy	ADE	0.157	(.03, .29)	0.00	(.00, .00)	0.16	(.03, .29)	0.84	(.71, .97)	-2563.9	-2551.9			
	AE	0.148	(.02, .27)	0.15	(.02, .27)			0.85	(.73, .98)	-2563.7	-2553.7	0.2	1	0.664
	DE	0.157	(.03, .29)			0.16	(.03, .29)	0.84	(.71, .97)	-2563.9	-2553.9	0.2	1	0.664
Transition Probability	ACE	0	(.00, .00)	0.00	(.00, .00)	0.09	(.02, .20)	0.91	(.80, 1.02)	-2024.1	-2012.1			
	AE	0.088	(-.03, .21)	0.09	(.03, .21)			0.91	(.79, 1.03)	-2023.3	-2013.3	0.7	1	0.387
	CE	0	(.00, .00)			0.09	(.02, .20)	0.91	(.80, 1.02)	-2024.1	-2014.1	0.7	1	0.387
Modularity$_{\text{Time-Varying}}$	ACE	0	(.00, .00)	0.00	(.00, .00)	0.05	(.06, .16)	0.95	(.84, 1.06)	-14.6	-128.6			
	AE	0.034	(-.10, .16)	0.03	(.10, .16)			0.97	(.84, 1.10)	-14.2	-13.2	0.4	1	0.523
	CE	0	(.00, .00)			0.05	(.06, .16)	0.95	(.84, 1.06)	-14.6	-13.6	0.4	1	0.523
FC$_{\text{Time-Varying}}$ of data-driven clusters (4%)	ADE	0.035	(-.05, .11)	0.00	(.00, .00)	0.04	(.05, .11)	0.97	(.89, 1.05)	-7003.4	-6991.4			
	AE	0.031	(-.05, .11)	0.03	(.05, .11)			0.97	(.89, 1.05)	-7003.4	-6993.4	0.0	1	0.838
	DE	0.035	(-.05, .11)			0.04	(.05, .11)	0.97	(.89, 1.05)	-7003.4	-6993.4	0.0	1	0.838
**Theta band**														
Fractional Occupancy	ACE	0.166	(-.21, .54)	0.17	(.21, .54)	0.05	(.27, .37)	0.79	(.66, .91)	-2493.0	-249.0			
	AE	0.219	(.10, .34)	0.22	(.10, .34)			0.78	(.66, .90)	-2492.9	-2482.9	0.1	1	0.804
	CE	0	(.00, .00)			0.18	(.08, .29)	0.82	(.71, .92)	-2492.4	-2482.4	0.1	1	0.804
Transition Probability	ACE	0.071	(-.33, .47)	0.07	(.33, .47)	0.04	(.29, .38)	0.89	(.75, 1.02)	-1584.1	-1572.1			
	AE	0.121	(-.01, .25)	0.12	(.01, .25)			0.88	(.75, 1.01)	-1584.0	-1574.0	0.1	1	0.82
	CE	0	(.00, .00)			0.10	(.01, .21)	0.90	(.79, 1.01)	-1584.0	-1574.0	0.1	1	0.82
Modularity$_{\text{Time-Varying}}$	ACE	0	(.00, .00)	0.00	(.00, .00)	0.02	(.09, .13)	0.98	(.87, 1.09)	145.6	157.6			
	AE	0.003	(-.12, .12)	0.00	(.12, .12)			1.00	(.88, 1.12)	145.7	155.7	0.1	1	0.78
	CE	0	(.00, .00)			0.02	(.09, .13)	0.98	(.87, 1.09)	145.6	155.6	0.1	1	0.78
FC$_{\text{Time-Varying}}$ of data-driven clusters (4%)	ACE	0	(-.00, .00)	0.00	(.00, .00)	0.05	(.04, .13)	0.96	(.87, 1.04)	-6897.2	-6885.2			
	AE	0.039	(-.04, .12)	0.04	(.04, .12)			0.96	(.88, 1.04)	-6897.0	-6887.0	0.2	1	0.685
	CE	0	(.00, .00)			0.05	(.03, .13)	0.96	(.87, 1.03)	-6897.2	-6887.2	0.2	1	0.685
**Alpha band**														
Fractional Occupancy	ACE	0	(.00, .00)	0.00	(.00, .00)	0.00	(.00, .00)	1.00	(1.00, 1.00)	-1903.0	-1891.0			
	AE	0	(.00, .00)	0.00	(.00, .00)			1.00	(1.00, 1.00)	-1903.0	-1893.0	6.8	1	1
	CE	0	(.00, .00)			0.00	(.00, .00)	1.00	(1.00, 1.00)	-1903.0	-1893.0	6.8	1	1
Transition Probability	ACE	0.347	(-.01, .71)	0.35	(.01, .71)	0.18	(.15, .51)	0.47	(.38, .57)	-1969.3	-1957.3			
	AE	0.534	(.45, .62)	0.53	(.45, .62)			0.47	(.38, .55)	-1968.4	-1958.4	0.9	1	0.331
	CE	0	(.00, .00)			0.47	(.39, .56)	0.53	(.44, .61)	-1965.4	-1955.4	0.9	1	0.331

Modularity$_{Time\text{-}Varying}$	ADE	0.129	(-.00, .26)	0.00	(.00, .00)	0.13	(.00, .26)	0.87	(.74, 1.00)	672.9	684.9			
	AE	0.12	(-.01, .25)	0.12	(.01, .25)			0.88	(.75, 1.01)	673.1	683.1	0.2	1	0.654
	DE	0.129	(-.00, .26)			0.13	(.00, .26)	0.87	(.74, 1.00)	672.9	682.9	0.2	1	0.654
FC$_{Time\text{-}Varying}$ of data-driven clusters (4%)	ADE	0.075	(-.03, .18)	0.00	(.00, .00)	0.08	(.03, .18)	0.93	(.82, 1.03)	-6546.7	-6534.7			
	AE	0.064	(-.03, .16)	0.06	(.03, .16)			0.94	(.84, 1.03)	-6546.3	-6536.3	0.3	1	0.562
	DE	0.075	(-.02, .17)			0.08	(.02, .17)	0.93	(.83, 1.02)	-6546.7	-6536.7	0.3	1	0.562
**Beta band**														
Fractional Occupancy	ADE	0.307	(.17, .44)	0.10	(.43, .62)	0.21	(.35, .77)	0.69	(.56, .83)	-2238.7	-2226.7			
	AE	0.289	(.16, .42)	0.29	(.16, .42)			0.71	(.58, .84)	-2238.3	-2228.3	0.4	1	0.539
	DE	0.311	(.18, .44)			0.31	(.18, .44)	0.69	(.56, .82)	-2238.6	-2228.6	0.4	1	0.539
Transition Probability	ADE	0.198	(.05, .34)	0.00	(.00, .00)	0.20	(.05, .34)	0.80	(.66, .95)	-2204.5	-2192.5			
	AE	0.166	(.03, .30)	0.17	(.03, .30)			0.83	(.70, .97)	-2203.3	-2193.3	1.2	1	0.268
	DE	0.198	(.05, .34)			0.20	(.05, .34)	0.80	(.66, .95)	-2204.5	-2194.5	1.2	1	0.268
Modularity$_{Time\text{-}Varying}$	ACE	0.017	(-.42, .45)	0.02	(.42, .45)	0.09	(.29, .46)	0.89	(.76, 1.03)	515.9	527.9			
	AE	0.117	(-.01, .24)	0.12	(.01, .24)			0.88	(.76, 1.01)	516.1	526.1	0.2	1	0.654
	CE	0	(.00, .00)			0.10	(.01, .21)	0.90	(.79, 1.01)	515.9	525.9	0.2	1	0.654
FC$_{Time\text{-}Varying}$ of data-driven clusters (4%)	ADE	0.124	(.01, .24)	0.00	(.00, .00)	0.12	(.01, .24)	0.88	(.76, .99)	-6879.2	-6867.2			
	AE	0.105	(-.00, .21)	0.11	(.00, .21)			0.90	(.79, 1.00)	-6878.5	-6868.5	0.7	1	0.394
	DE	0.124	(.01, .23)			0.12	(.01, .23)	0.88	(.77, .99)	-6879.2	-6869.2	0.7	1	0.394
**Gamma band**														
Fractional Occupancy	ADE	0.205	(.06, .35)	0.00	(.00, .00)	0.21	(.06, .35)	0.80	(.65, .94)	-1886.5	-1874.5			
	AE	0.174	(.03, .31)	0.17	(.03, .31)			0.83	(.69, .97)	-1885.6	-1875.6	0.9	1	0.33
	DE	0.205	(.06, .35)			0.21	(.06, .35)	0.80	(.65, .94)	-1886.5	-1876.5	0.9	1	0.33
Transition Probability	ADE	0.318	(.20, .44)	0.00	(.00, .00)	0.32	(.20, .44)	0.68	(.56, .80)	-1581.9	-1569.9			
	AE	0.298	(.18, .41)	0.30	(.18, .41)			0.70	(.59, .82)	-158.2	-157.2	1.6	1	0.202
	DE	0.318	(.20, .44)			0.32	(.20, .44)	0.68	(.56, .80)	-1581.9	-1571.9	1.6	1	0.202
Modularity$_{Time\text{-}Varying}$	ADE	0.012	(-.13, .15)	0.00	(.00, .00)	0.01	(.13, .15)	0.99	(.85, 1.13)	659.0	671.0			
	AE	0	(.00, .00)	0.00	(.00, .00)			1.00	(1.00, 1.00)	659.1	669.1	0.0	1	0.86
	DE	0.012	(-.13, .15)			0.01	(.13, .15)	0.99	(.85, 1.13)	659.0	669.0	0.0	1	0.86
FC$_{Time\text{-}Varying}$ of data-driven clusters (4%)	ACE	0	(.00, .00)	0.00	(.00, .00)	0.00	(.00, .00)	1.00	(1.00, 1.00)	-6539.5	-6527.5			
	AE	0	(.00, .00)	1.00	(1.00, 1.00)			1.00	(1.00, 1.00)	-6539.5	-6529.5	7.9	9	1
	CE	0	(.00, .00)			0.00	(.00, .00)	1.00	(1.00, 1.00)	-6539.5	-6529.5	7.9	9	1

All models were adjusted for age and sex. A, Additive genetic effect; C, common environmental effect; D, dominant genetic effect; E, Unique/non-shared environment effect; -2LL, twice the negative log-likelihood; AIC, Akaike's information criterion; df, degrees of freedom; χ^2, chi square, Δdf, change in degree of freedom between the full model and the nested model; *p*, χ^2 test in model fitting. The

AE and CE models are nested within the ACE model. Each nested model is compared with the fully saturated model. The fitness of models was tested based on a change in AIC (for a change of df of 1, the statistically significant change in χ^2 is 3.84). h^2, the narrow-sense heritability estimated as $\sigma^2_A/(\sigma^2_A + \sigma^2_c + \sigma^2_E)$; the broad-sense heritability estimated as $(\sigma^2_A + \sigma^2_D)/(\sigma^2_A + \sigma^2_D + \sigma^2_E)$; CI: Confidence Interval (lower bound, upper bound).

Supplementary Figures

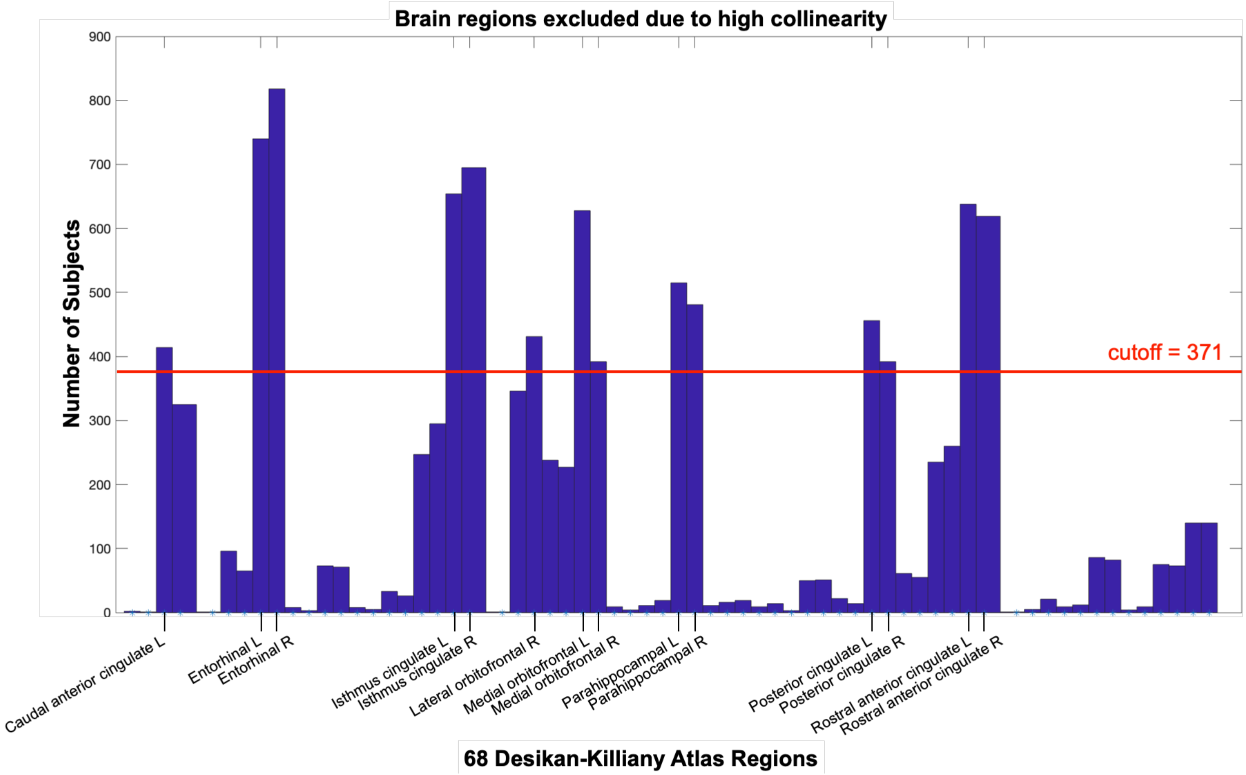

Figure S1. Top 14 regions with high collinearity. To mitigate the source-leakage confound, caused by the blurring of point dipole sources and the spreading of signals across neighboring regions, we excluded regions, whose extracted signals were found to be highly collinear with others based on *qr* function in Matlab. Subsequently, we excluded 14 regions which displayed highly collinearity in over 40% of the 928 subjects (cutoff = 371) from the investigation: bilateral '*rostral anterior cingulate'*, bilateral '*posterior cingulate*', bilateral '*parahippocampal*', bilateral '*medial orbitofrontal'*, bilateral '*isthmus cingulate*', bilateral '*entorhinal*', '*lateral orbitofrontal R*', and '*caudal anterior cingulate L'*.

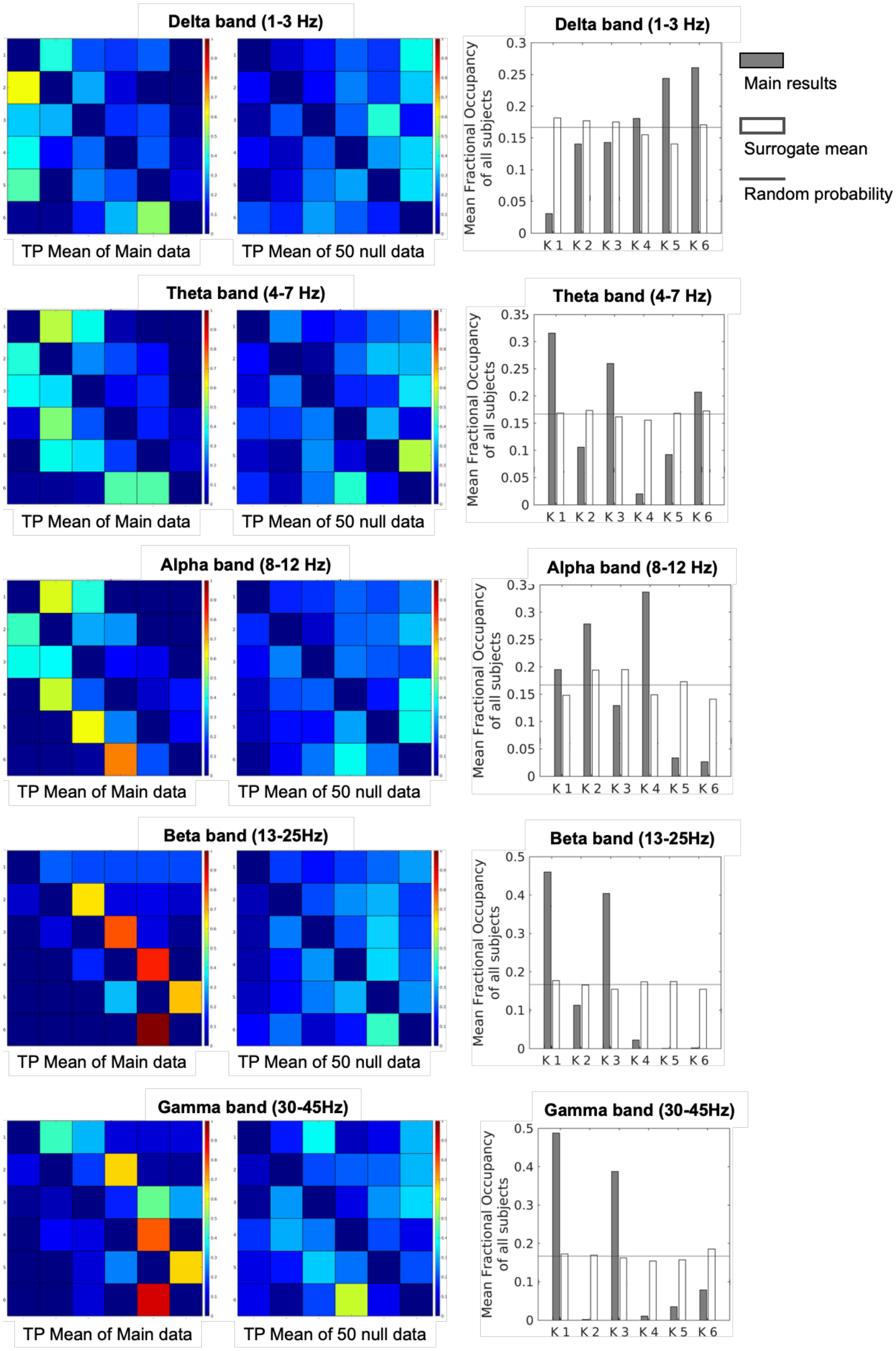

Figure S2. Difference in Transition Probability and Fractional Occupancy distribution obtained at *K* of 6 between main dataset and 50 surrogate datasets. Fractional Occupancy and Transition Probability matrix averaged from the 50 surrogate data show little structured distribution across states (or state pairs), indicating the absence of meaningful dynamic properties in the surrogate

data, whereas Fractional Occupancy and Transition Probability obtained from the real data show non-random sequencing of brain states.

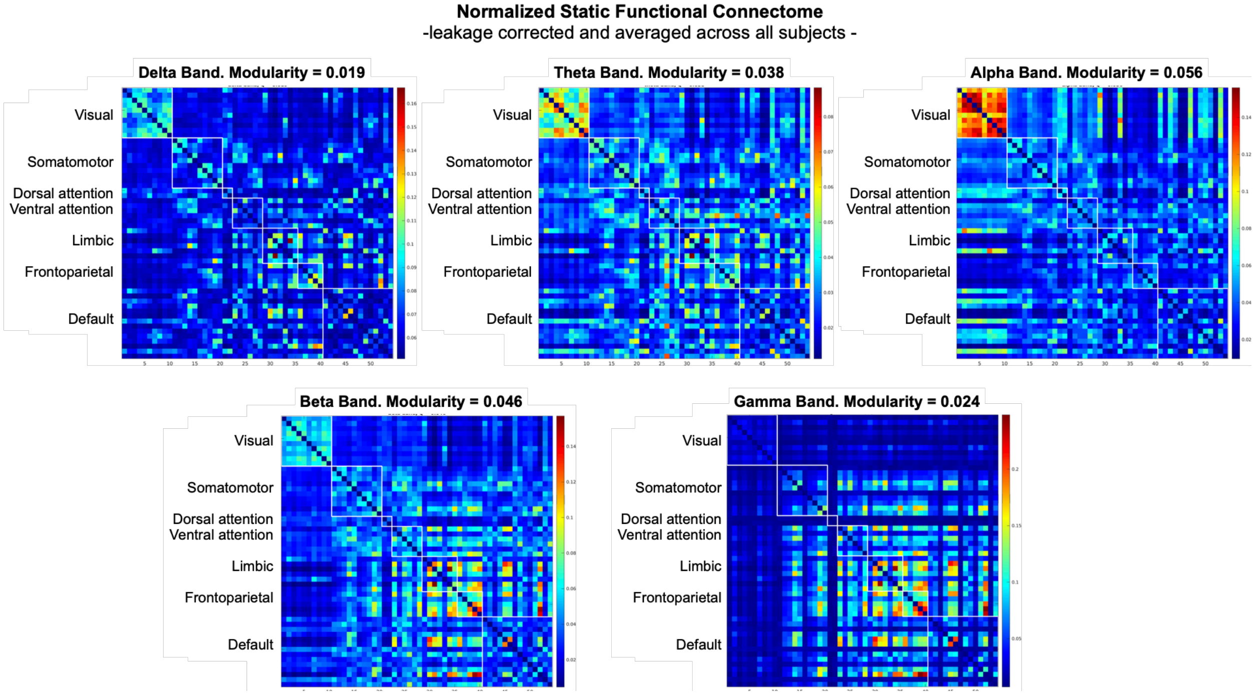

Figure S3. Static functional connectome for each canonical band averaged across all subjects in the study. The 54 distinct regions of Desikan-Killiany Atlas, excluding 14 regions with high collinearity. The regional signals underwent detrending and bandpass filtering within canonical frequency ranges: delta (1-3 Hz), theta (4-7 Hz), alpha (8-12 Hz), beta (13-25 Hz), and gamma (30-45 Hz). Subsequently, we used a symmetric orthogonalization procedure (Colclough et al., 2015) to remove all shared signal at zero lag between the regions. Functional connectivity for each pair-wise connection was calculated as Pearson correlation between the two amplitude envelopes. Finally, the functional connectivity matrix was normalized using Fisher's r-to-z transformation.

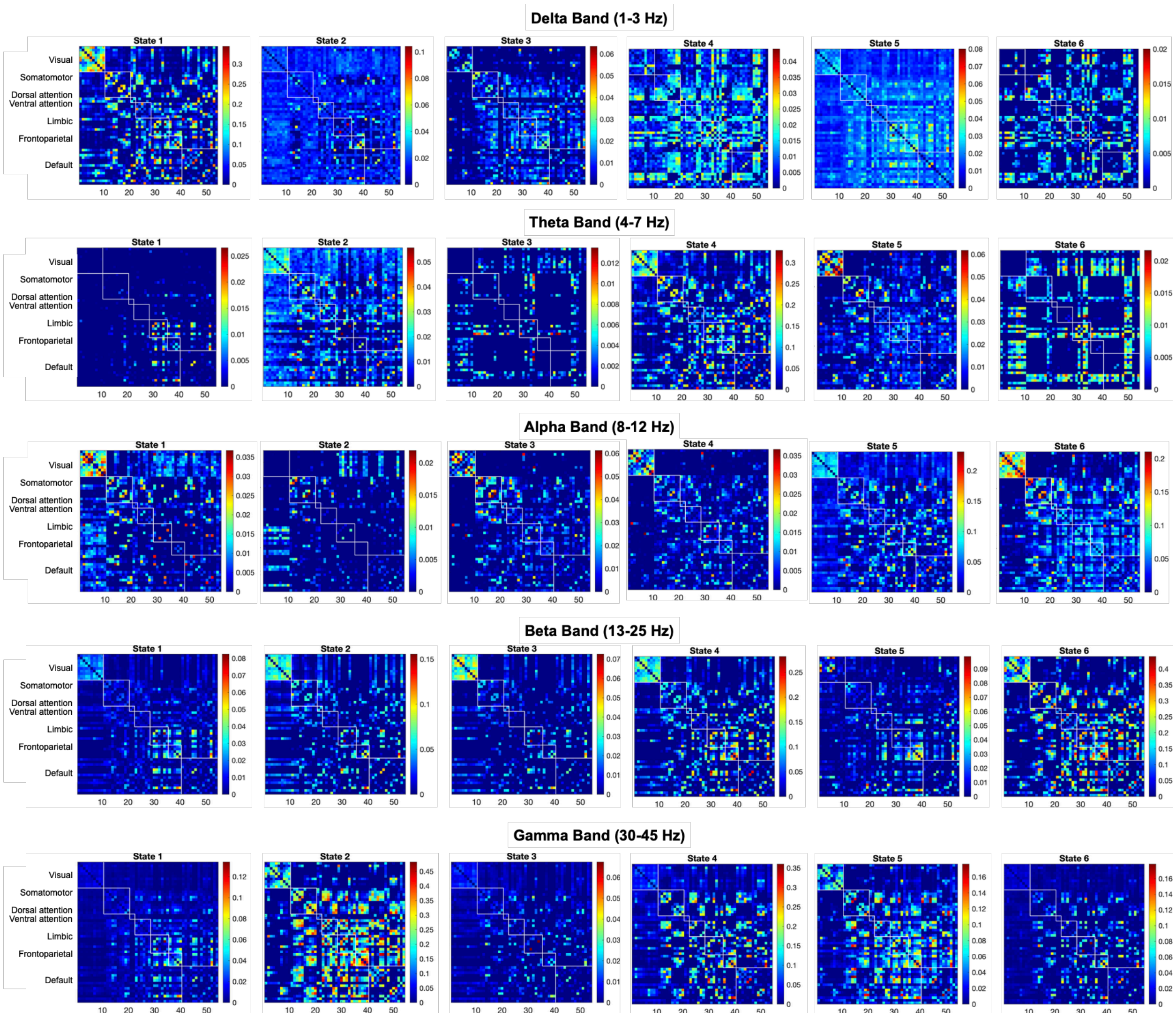

Figure S4. Distinct HMM connectome states for each canonical band. HMM estimates the connectome states from the leakage-corrected EEG signals that are common to all subjects. The states are represented by z-scored functional connectivity (FC) matrices. The rows and columns represent 54 regions organized according to their membership to canonical intrinsic connectivity networks (ICNs) (Yeo et al., 2011).

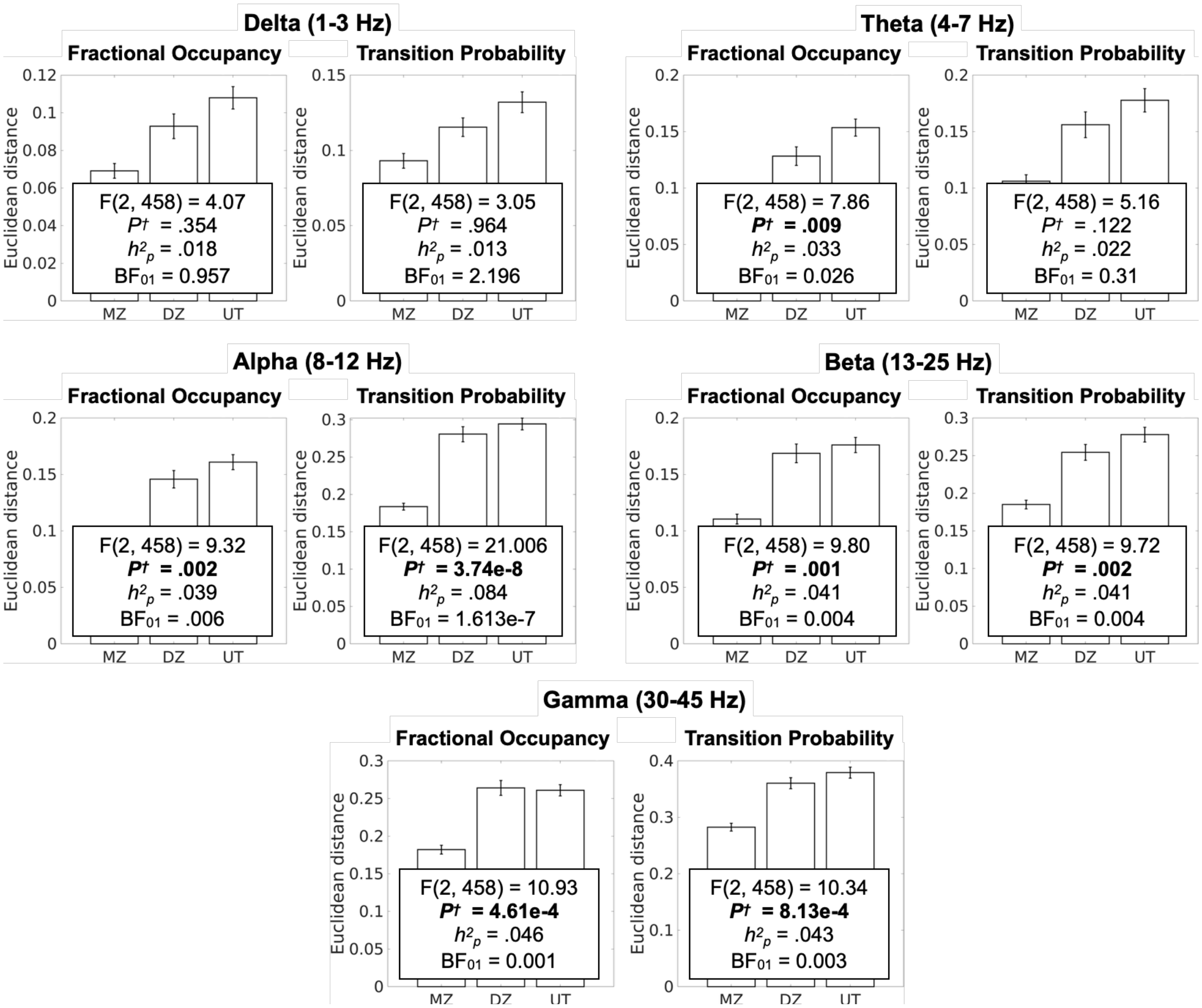

Figure S5. Heritability of temporal features of the dynamic connectome (*K* = 4). Heritability of each of the multivariate features was assessed separately by one-way ANCOVAs of the factor sibling status (three levels: monozygotic twins (MZ), sex-matched dizygotic twins (DZ), and sex-matched pairs of unrelated individuals), adjusted for age and sex. The main effect of sibling status indicates the heritability, or genetic effect. (A) Bar graphs show that more genetically similar subject pairs have smaller Euclidean distance, which denotes similarity of a given temporal connectome feature. Specifically, temporal connectome features were more similar among MZ twin pairs than DZ twins, followed by pairs of unrelated individuals. $P^{\dagger}$: P values Bonferroni-corrected for 20 tests (four dependent variables and five frequency bands), h^2_p: Partial eta squared effect size.

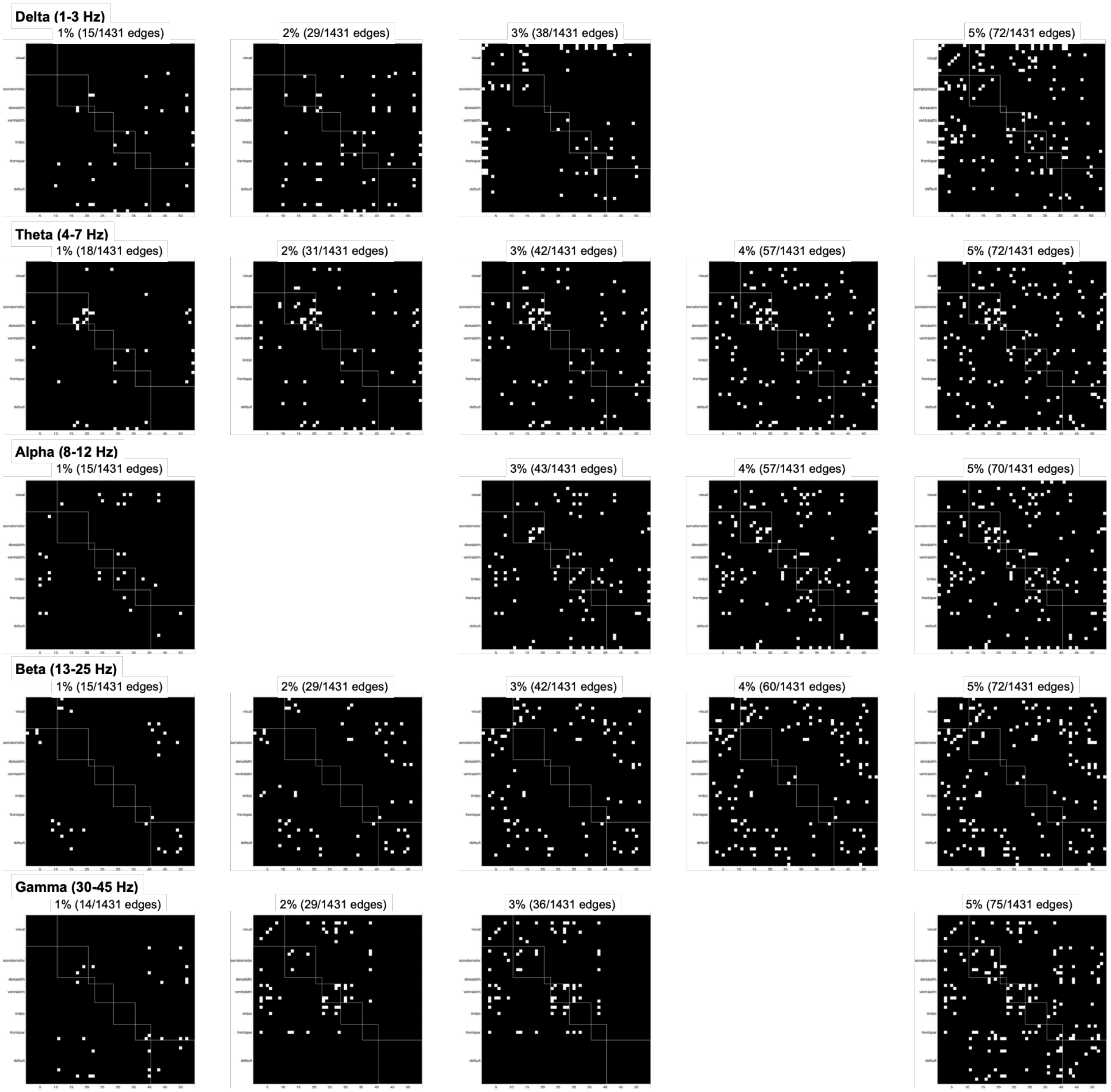

Figure S6. Binary NBS (data-driven) connectivity patterns. We performed network-based statistics (NBS) to select connected sets of connections (i.e., clusters), in a data-driven manner for subsequent heritability analysis. We started with the absolute functional connectivity (FC) matrices, where all connections were transformed into absolute values to focus on the strength of connections, regardless of their connectivity direction. For each connection, an ANCOVA of the factor state was calculated to quantify the degree to which each connection's connectivity strength differed across states, adjusted for age and sex. The ensuing connection-wise F statistics was threshold at arbitrary values, allowing 1 ~ 5% of connections to survive. NBS was applied to the threshold matrices, resulting in one significant cluster at each threshold visualized as symmetric binary matrices. We averaged the absolute FC values of all connections in the cluster for each state, together constructing the multivariate spatial feature.
